# SARS-CoV-2 nucleocapsid protein hijacks host transcription by targeting eEF1A1

**DOI:** 10.26508/lsa.202603751

**Published:** 2026-10-14

**Authors:** Baptiste Bidon, Yu-Lin Lai, Tai-Ling Chao, Gawa Chen, Yee-Shin Lee, Hsiang-Hung Huang, Jia-Syuan Chen, Alejandro Gonzalez Losada, Sui-Yuan Chang, Jean-Marc Egly, Tsai-Kun Li

**Affiliations:** 1 Taiwan International Bio Research Center (TIBRC), College of Medicine, National Taiwan University , Taipei, Taiwan; 2 Department of Clinical Laboratory Sciences and Medical Biotechnology, College of Medicine, National Taiwan University , Taipei, Taiwan; 3 Department of Laboratory Medicine, National Taiwan University Hospital, Taipei, Taiwan; 4 Department of Life Science, Institute of Ecology and Evolutionary Biology, National Taiwan University , Taipei, Taiwan; 5 Cell Biology and Pharmacogenomics Department, PharmaMar S.A, Colmenar Viejo, Spain; 6 Institut de Génétique, Biologie Moléculaire et Cellulaire, Strasbourg (IGBMC), Université de Strasbourg, Strasbourg, France; 7 Development Center for Biotechnology, Taipei, Taiwan

## Abstract

SARS-CoV-2 variants display substantial genetic and phenotypic diversity, yet the molecular mechanisms underlying their effects on host gene regulation need further investigation. We performed a transcriptomic analysis of host responses to multiple SARS-CoV-2 variants, including Delta and Omicron. Despite their divergence, all variants induced a conserved transcriptional program in human lung epithelial cells characterized by activation of interferon regulatory factors (IRFs), immediate–early genes, and downstream cytokines. We identify the viral nucleocapsid (N) protein as a central driver of this transcriptional reprogramming, as N expression alone partially recapitulated the host response to infection. Mechanistically, N promotes recruitment of interferon regulatory factors and AP-1 transcription factors, RNA polymerase II, and the elongation factor eEF1A1 to promoters of pro-inflammatory genes, revealing a previously unrecognized nuclear role for eEF1A1. Disruption of the N-eEF1A1 interaction, genetically or pharmacologically using the eEF1A interactant plitidepsin, abolished transcriptional complex formation and reversed host gene dysregulation, identifying a conserved host–virus regulatory axis and potential pan-variant therapeutic target.

## Abstract (precis)

SARS-CoV-2 nucleocapsid promotes recruitment of IRF and AP-1 transcription factors, RNA polymerase II, and the elongation factor eEF1A1 to promoters of pro-inflammatory genes, revealing a previously unrecognized nuclear role for eEF1A1.

## Introduction

RNA viruses are the primary drivers of recent outbreaks and emerging infectious diseases, including HIV, influenza, Zika, Ebola, and SARS-CoV-2 (Mohanty et al, 2023). Since 2019, COVID-19 has caused more than 7.1 million deaths worldwide (https://www.who.int/news-room/fact-sheets/detail/coronavirus-disease-(covid-19)), although mortality rates have declined after June 2022 (https://www.who.int/). Because of their rapid evolutionary dynamics, RNA viruses continuously generate variants with altered transmissibility, infectivity, and pathogenicity. SARS-CoV-2 illustrates this challenge as its evolving variants continue to pose a significant global health threat. Variant’s impact depends not only on viral evolution but also on vaccination coverage, therapeutic interventions, and host health status (Markov et al, 2023). For example, the Delta variant was associated with higher mortality than Omicron, particularly in adults (Sigal et al, 2022; Liu et al, 2023). Although the original Omicron (BA.1) lineage has largely disappeared, several subvariants including XFG, NB.1.8.1, JN.1, LP.8.1 KP.3.1.1, and XEC continue to circulate globally.

SARS-CoV-2 depends on host cellular machinery to replicate and propagate. Consequently, therapeutic strategies target either viral components or host pathways exploited by the virus (Ray et al, 2020; Kumar et al, 2021). Additional approaches aim to limit pathological host responses, such as cytokine storm, which are associated with elevated levels of inflammatory mediators including IL-6 and IL-10, important biomarkers of disease severity (Hojyo et al, 2020).

The SARS-CoV-2 genome encodes several proteins essential for replication, including RNA-dependent RNA polymerase, the main protease (Mpro), and structural proteins such as spike (S), nucleocapsid (N), membrane (M), and envelope (E) (Baggen et al, 2021). Much research has focused on S, which mediates viral entry through the ACE2 receptor (Cheng et al, 2020; Harvey et al, 2021; Huang et al, 2021; Chang et al, 2022). However, rapid *S* gene evolution raises concerns regarding vaccine protection and long-term immunity, highlighting the need for alternative therapeutic targets.

Recent virus–host interactome studies and drug repurposing screens have identified host translation machinery as a key vulnerability exploited by SARS-CoV-2 (Gordon et al, 2020; Riva et al, 2020; Molina et al, 2025). In particular, the viral N protein interferes with host translation machinery through different proteins such as the ribosomal protein L5 and the elongation factor eEF1A (Zhu et al, 2021; Molina et al, 2025; Wang et al, 2025).

eEF1A is one of the most abundant cellular proteins and plays a role in translation elongation by delivering aminoacyl-tRNAs to the ribosome. Beyond translation, it also participates in nuclear export, cytoskeletal organization, protein degradation, and transcriptional regulation (Khacho et al, 2008; Zúñiga et al, 2010; Vera et al, 2014).

The N protein is a multifunctional regulator during early infection (McBride et al, 2014; Peng et al, 2020). It binds viral genomic RNA to form ribonucleoprotein complexes required for viral transcription and replication. It can also modulate host immune responses (Lu et al, 2011). Importantly, inhibitors targeting eEF1A show strong antiviral activity. Among them, plitidepsin (Aplidin, PharmaMar), a marine-derived compound originally developed as an anticancer drug, displays potent antiviral and anti-inflammatory properties (Lee et al, 2012; Rodon et al, 2021). Approved in Australia for relapsed multiple myeloma, plitidepsin inhibits the translation machinery and has shown promising activity in clear-cell ovarian cancer (Losada et al, 2016; Miyake et al, 2021), and against SARS-CoV-2 (White et al, 2021). However, the molecular mechanisms by which the SARS-CoV-2 N protein alters host cell biology, and how targeting eEF1A with plitidepsin could counteract these effects, have not been thoroughly investigated yet.

In this study, we analyzed viral infectivity and host transcriptional responses using a panel of representative SARS-CoV-2 variants in human Calu-3 cells. RNA-sequencing (RNA-seq) analyses were performed before and after infection to identify shared and variant-specific host transcriptional responses. In parallel, cells were transfected with the viral N protein to directly assess its role in host gene expression reprogramming. After identification of induced regulators such as immediate–early genes (IEGs) and interferon regulatory factors (IRFs), we investigated their role in activating host-responsive genes, particularly cytokines. Using ChIP-qPCR, we investigated whether the N protein, together with IRFs and/or IEGs, eEF1A1, and RNA polymerase II (Pol II), binds to the promoters of pro-inflammatory genes and contributes to their transcriptional activation. We further dissected the molecular interaction between eEF1A1 and N protein and determined whether disruption of this interaction using either plitidepsin or a competitive peptide restores normal host gene expression and reduces viral infectivity across multiple variants. Collectively, this study helped to clarify the role of the N protein in SARS-CoV-2 infectivity and defined its functional interplay with eEF1A1 as a key driver of virus-induced transcriptional reprogramming, highlighting a potential therapeutic target.

## Results

### Transcriptional responses to SARS-CoV-2 variants and their reversal by plitidepsin

**Figure S1.**
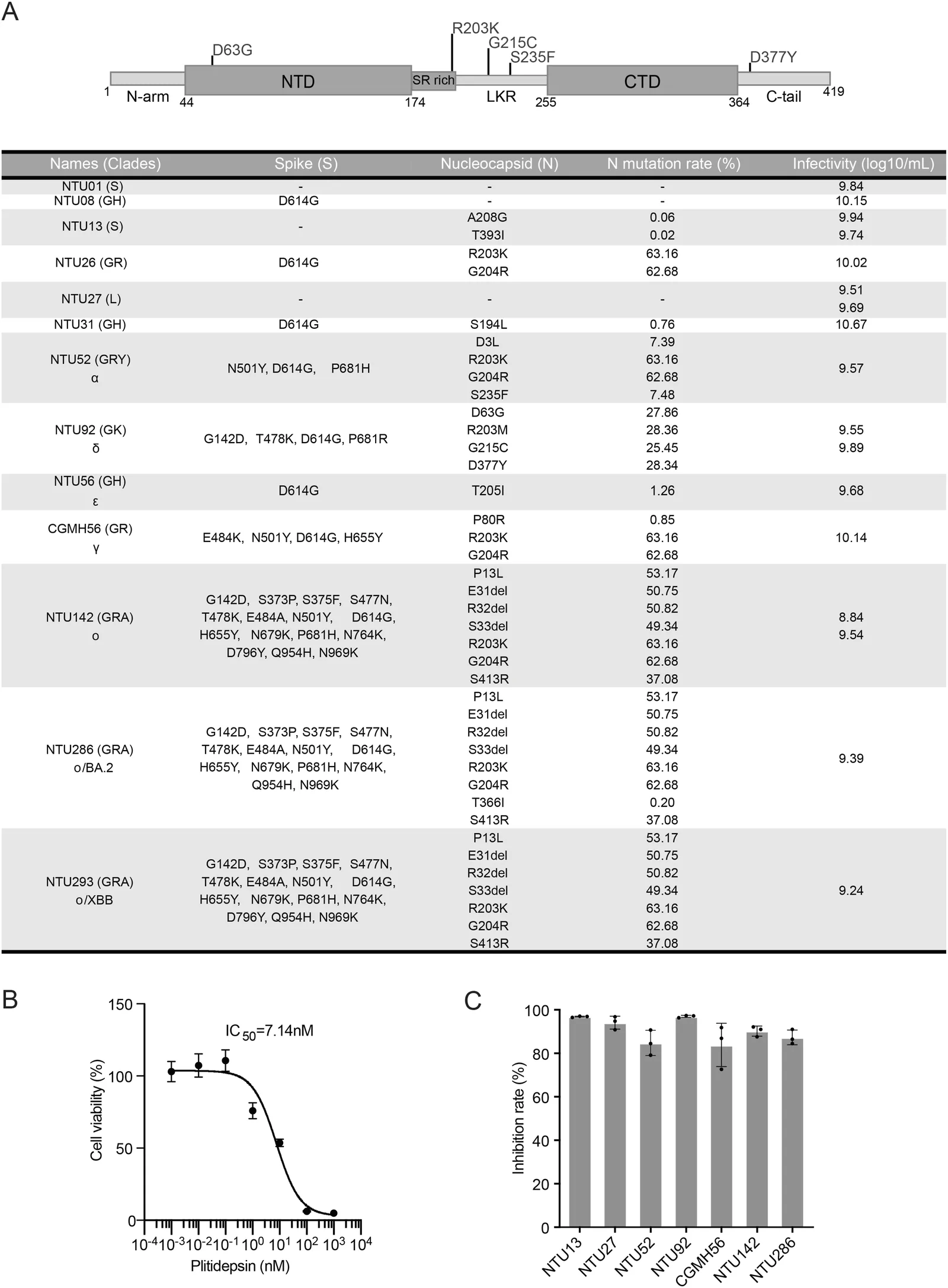
SARS-CoV-2 variants sensitivity. **(A)** Structure of the SARS-CoV-2 N protein, with N-arm, NTD as a RNA-binding domain, SR-rich region, LKR, CTD as a dimerization domain, and C-tail. Mutation sites in the N protein are indicated. Table of variant strains with mutation either in S or in N protein, classified by GISAID clades. Viruses were isolated and named by National Taiwan University and Chang Gung Memorial Hospital in Taiwan. The percentage of SARS-CoV-2 N protein mutations in the context of variants represented in the National Taiwan University/Chang Gung Memorial Hospital collection. Infectivity is determined by the copy numbers of SARS-CoV-2 variant strain–infected Calu-3 cells. **(B)** IC_50_ evaluation of plitidepsin. Plaque reduction assay performed in Calu-3 cells, 24 h after NTU13 infection treated with increasing concentrations of plitidepsin. The x axis indicates plitidepsin concentration, and the y axis shows the percentage of plaque numbers relative to the untreated control. Standard deviations are depicted. **(C)** Yield reduction assay of virus productive inhibition in Calu-3 cells infected by several SARS-CoV-2 viruses further treated by 20 nM plitidepsin, as indicated at the bottom of the panel. NTD, N-terminal domain; LKR, linker region; CTD, C-terminal domain.

We first collected multiple SARS-CoV-2 variants circulating in Taiwan with mutations in the viral N protein, including NTU52(α), NTU56 (ε), NTU92(δ), CGMH56(γ), NTU142(ο), NTU286(ο/BA.2), NTU293(ο/XBB), NTU13, and one original Wuhan strain NTU27 carrying the wild-type (WT) *N* gene (Fig S1A) and showing similar infectivity, ranging from 9.24 log_10_/ml (NTU293) to 10.34 log_10_/ml (CGMH56). Plitidepsin significantly reduced viral infection (Martinez, 2021; White et al, 2021). In NTU13-infected Calu-3 cells, the IC_50_ was 7.14, and 10 nM plitidepsin reduced infectious particles by >80% across all tested variants (Fig S1B and C). These findings demonstrate the broad-spectrum antiviral activity against SARS-CoV-2 variants independent of mutations in the N or S proteins.

**Figure 1.**
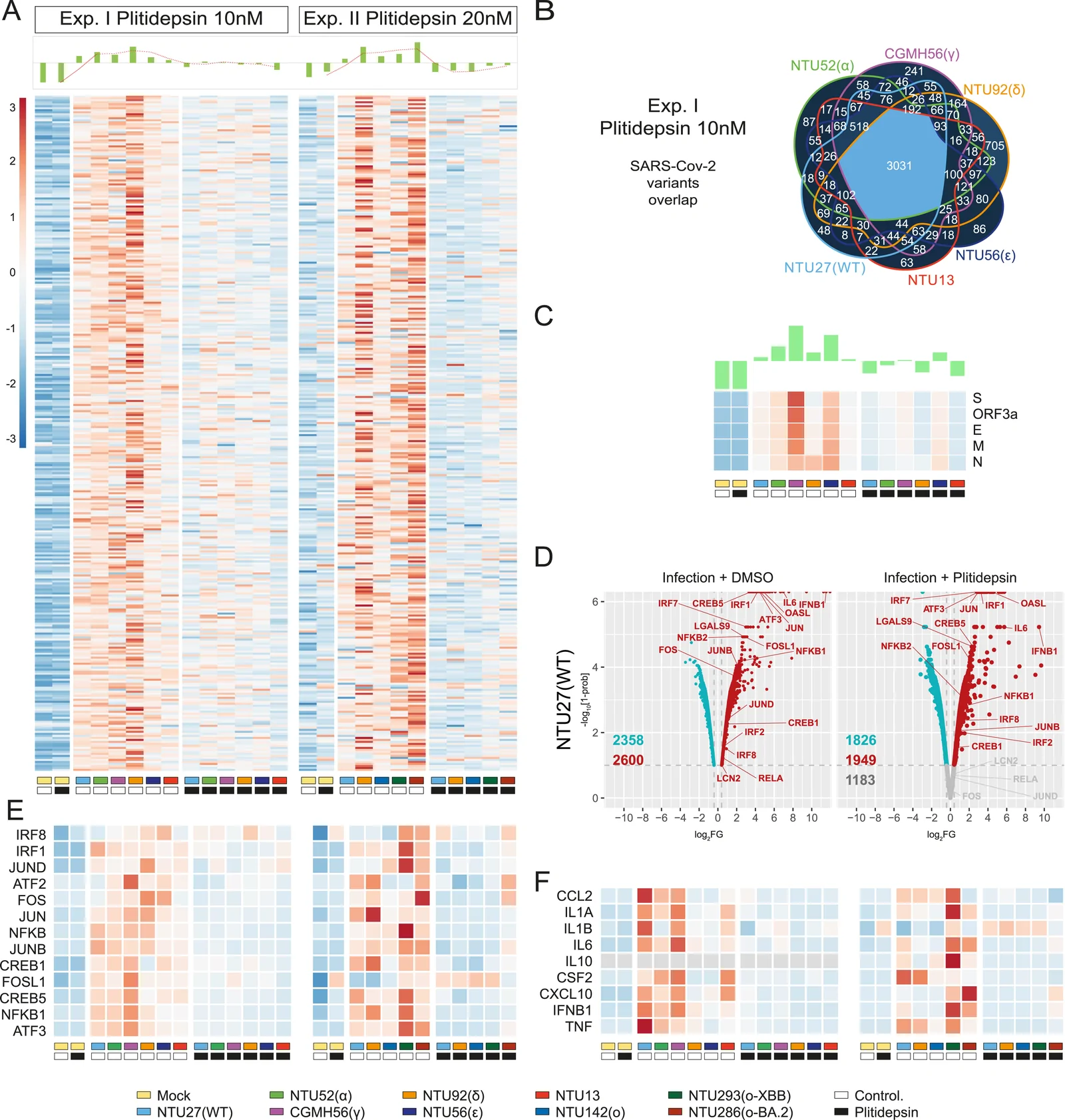
RNA expression profiling of Calu-3 cells infected by SARS-CoV-2 variants in the presence or absence of plitidepsin. **(A)** Heatmap of clustered genes in Calu-3 cells infected by the indicated SARS-CoV-2 variants (colored squares, bottom), before (white squares) and after (black squares) treatment with plitidepsin (10 nM in Experiment I and 20 nM in Experiment II). The moving average of expression for the whole cluster before and after treatment is shown at the top. **(B)** Venn diagram showing the overlap of dysregulated genes in Calu-3 cells infected with NTU52(α), CGMH56(γ), NTU92(δ), NTU56(ε), and NTU13 and NTU27(WT) variants. The value 3,031 denotes the number of common DEGs in Experiment I. **(C)** Heatmap showing the expression levels of the viral S, ORF3a, E, M, and N proteins in the corresponding infected Calu-3 cells further treated or not by plitidepsin from Experiment I, as indicated by the colored squares at the bottom of the panel. **(D) **Volcano plot showing DEGs (fold change > 2; Q-value = 0.9) in Calu-3 cells infected with the SARS-CoV-2 variants NTU27(WT) in the absence (left panels) or presence (right panels) of plitidepsin. Red and blue dots represent up-regulated and down-regulated genes, respectively, whereas gray dots indicate genes, whose expression is restored upon plitidepsin treatment. Selected genes whose expression varies depending on infection and plitidepsin treatment are indicated. **(E)** Heatmap showing RNA-seq expression levels of IEGs and IRFs in Calu-3 cells infected with SARS-CoV-2 variants, with or without plitidepsin treatment. **(F)** Heatmap showing RNA-seq expression levels of cytokine-related genes in Calu-3 cells infected with SARS-CoV-2 variants, with or without plitidepsin treatment. At the bottom of the panel, colored squares represent noninfected Calu-3 cells (Mock) and cells infected with the various NTU variants, either untreated or treated with plitidepsin (black and white squares, respectively).

RNA-seq was performed on Calu-3 cells infected with NTU27(WT), NTU52(α), NTU92(δ), NTU56(ε), CGMH56(γ), and NTU13 (Experiment I) and with NTU27(WT), NTU92(δ), NTU142(ο), NTU286(ο/BA2), and NTU293(ο/XBB) (Experiment II) in the presence or absence of 10 or 20 nM plitidepsin, respectively. We found that in our experimental conditions, infection resulted in 4,744–5,614 differentially expressed genes (DEGs) in Experiment I and 2,674–4,302 DEGs in Experiment II with approximately half of the genes up-regulated (Table 1). Among those genes, we identified a cluster of 337 genes, which is significantly up-regulated by SARS-CoV-2 infection across all tested strains (Fig 1A) and whose expression is restored almost to basal levels by plitidepsin in a dose-dependent manner. The higher the dose, the closer the gene expression is to the healthy state. In contrast to other host genes, these genes therefore appear to be directly regulated by plitidepsin in infected cells.

**Table 1.** Identification of DEGs after SARS-CoV-2 infection or N protein transfection and their restoration by plitidepsin.

SARS-CoV-2	N mutations	Infectivity	Infection		Infection + plitidepsin	
​	​	log10/ml	Dysregulated (total)	Up-regulated	Restored (total)	Restored (up-reg)
Exp. I Pliti. 10 nM	​	​	​	​	​	​
NTU27(WT)	​	9.57	4,958	2,600	1,183 (23,9%)	651 (25,0%)
NTU52(α)	R203K-G204R	9.57	5,343	2,751	1,514 (28,3%)	797 (29,0%)
CGMH56(γ)	R203K-G204R	10.14	5,583	2,846	1,168 (20,9%)	611 (21,5%)
NTU92(δ)	R203M-D377Y	9.72	5,614	2,810	1,993 (35,5%)	931 (33,1%)
NTU56 (ε)	T205I	9.68	5,133	2,636	1,259 (24,5%)	639 (24,2%)
NTU13	T391I-A208G	9.84	4,744	2,466	1,909 (40,2%)	1,000 (40,5%)
Exp. II Pliti. 20 nM	​	​	​	​	​	​
NTU27(WT)	–	​	3,258	1,965	1,559 (47,8%)	1,061 (54,0%)
NTU92(δ)	R203M-D377Y	9.72	4,302	2,251	2,454 (57.0%)	876 (38,9%)
NTU142(ο)	R203K-G204R	9.29	2,674	1,520	1,438 (53,7%)	885 (58,2%)
NTU286(ο,BA2)	E31del-R203K-G204R	9.39	3,894	2,149	1,830 (46,9%)	1,097 (51,0%)
NTU293(ο,XBB)	E31del-R203K-G204R	9.24	3,627	1,984	1,183 (32,6%)	622 (31,3%)

Viral N protein	​	​	Transfection		Transfection + plitidepsin	
N-WT	​	​	547	236	289 (52,8%)	151 (64%)
N-R203K	​	​	268	88	147 (54,0%)	59 (67.0%)
N-D377Y	​	​	437	173	222 (50,8%)	111 (64,2%)
N-del	​	​	796	351	388 (48,7%)	196 (55,8%)

Calu-3 cells were either infected with different SARS-CoV-2 variants or transfected with WT or mutant viral N proteins, followed by treatment with 10 or 20 nM plitidepsin as indicated. DEGs (total and up-regulated) were identified under each condition. The right columns show the percentage of genes restored after plitidepsin treatment, calculated relative to either the total number of dysregulated genes or the subset of up-regulated genes. The left panels indicate the absolute number of dysregulated genes, including the numbers of up-regulated and down-regulated genes whose expression is restored after plitidepsin treatment under both infection and transfection conditions. Infectivity of SARS-CoV-2 variants was assessed by quantifying SARS-CoV-2 E gene copy numbers in infected Calu-3 cells.

**Figure S2.**
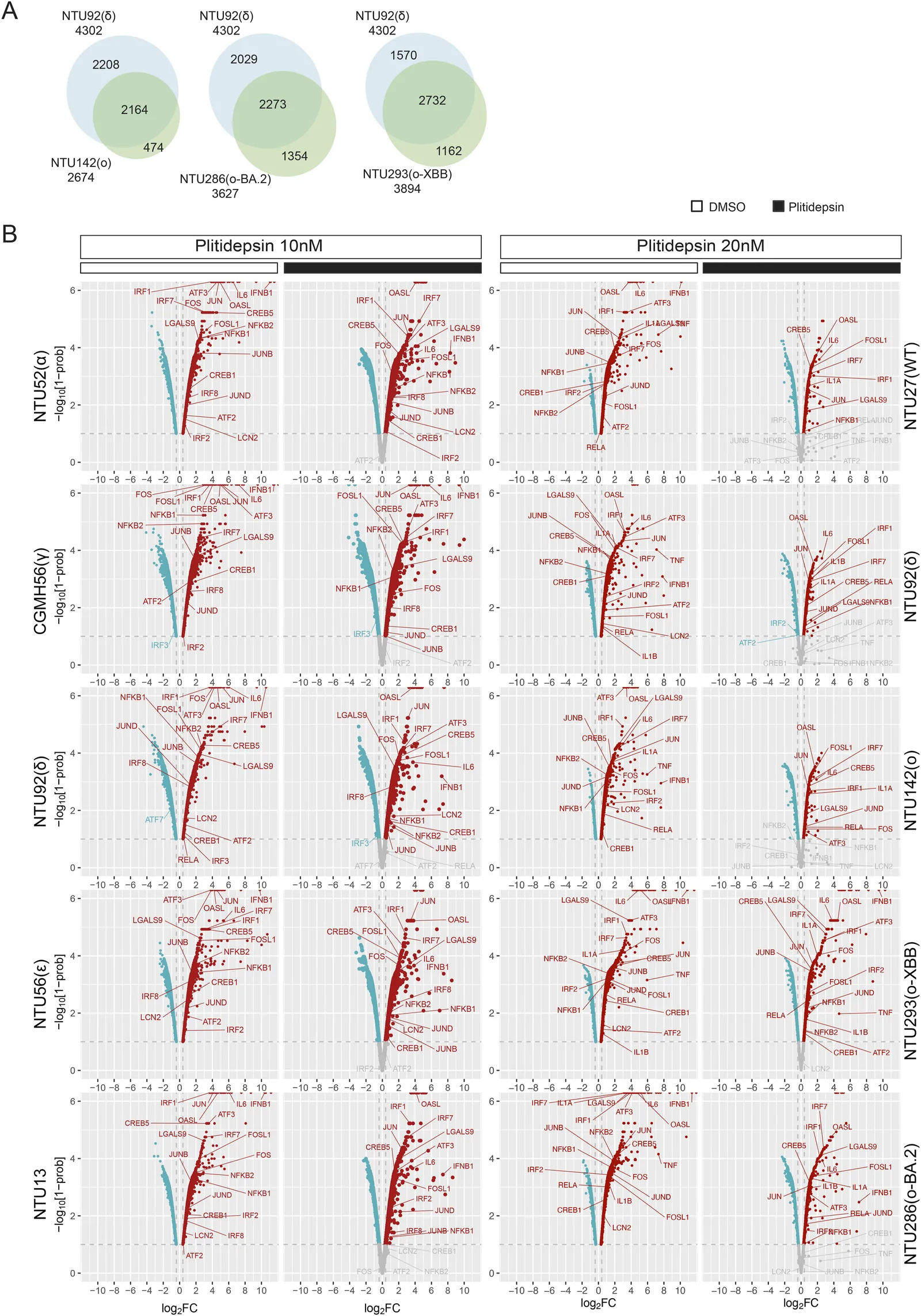
RNAseq on infected Calu-3 cells. **(A)** Venn diagram showing the overlap of dysregulated genes in Calu-3 cells infected with SARS-CoV-2 variants NTU92(δ) and omicron-associated NTU142(ο), NTU288(α/XBB), and NTU293(ο, XBB). **(B)** Volcano plot showing DEGs (fold change > 2; Q-value = 0.9) after infection of Calu-3 cells in Experiment I, with NTU52(α), CGMH56(γ), NTU92(δ), NTU56(ε), and NTU13 and in Experiment II with NTU27(WT), NTU92(δ), NTU142(ο), NTU288(α/XBB), and NTU293(ο, XBB) variants with (right panels) or without (left panel) 10 or 20 nM plitidepsin treatment, respectively. Red and blue dots represent up-regulated and down-regulated genes, respectively, whereas gray dots indicate genes, whose expression is restored upon plitidepsin treatment. Selected genes whose expression varies depending on infection and plitidepsin treatment are indicated.

Among all the identified DEGs, 3,031 genes were commonly altered across all NTU variants in Experiment I (Fig 1B). Notably, early Omicron caused a milder transcriptional response than NTU92(δ) (2,674 genes, Fig S2A), whereas later subvariants NTU293(ο/XBB) and NTU286(ο/BA.2) showed stronger activation (3,627 and 3,894 genes), reflecting modification in clinical severity (Wolter et al, 2022). Analysis of viral gene expression further revealed that viral RNA levels appeared to correlate with the severity of the associated symptoms. Importantly, treatment with plitidepsin markedly reduced the overall abundance of viral RNA in infected cells, consistent with its potent inhibitory effect on viral replication (Fig 1C).

Volcano plots illustrated DEGs detected after infection of Calu-3 cells in the absence (left panels) or presence (right panels) of 10 nM plitidepsin (Fig 1D). Down-regulated and up-regulated genes are indicated in blue and red, respectively. Up to 25% of DEGs by SARS-CoV-2 infection returned to baseline expression levels in NTU27(WT)-infected cells (Table 1 and Fig 1D, number in gray). Similarly, volcano plots of the 10 additional Calu-3 NTU variant–infected cells also revealed a marked shift in the expression of key up-regulated genes after treatment, including *IRFs*, *IEGs* (*FOS*/*JUN*), and pro-inflammatory genes (*IL6*, *IFNB*, and Oligoadenylate Synthetase-Like [*OASL*]) (Figs 1C and S2B).

**Figure S3.**
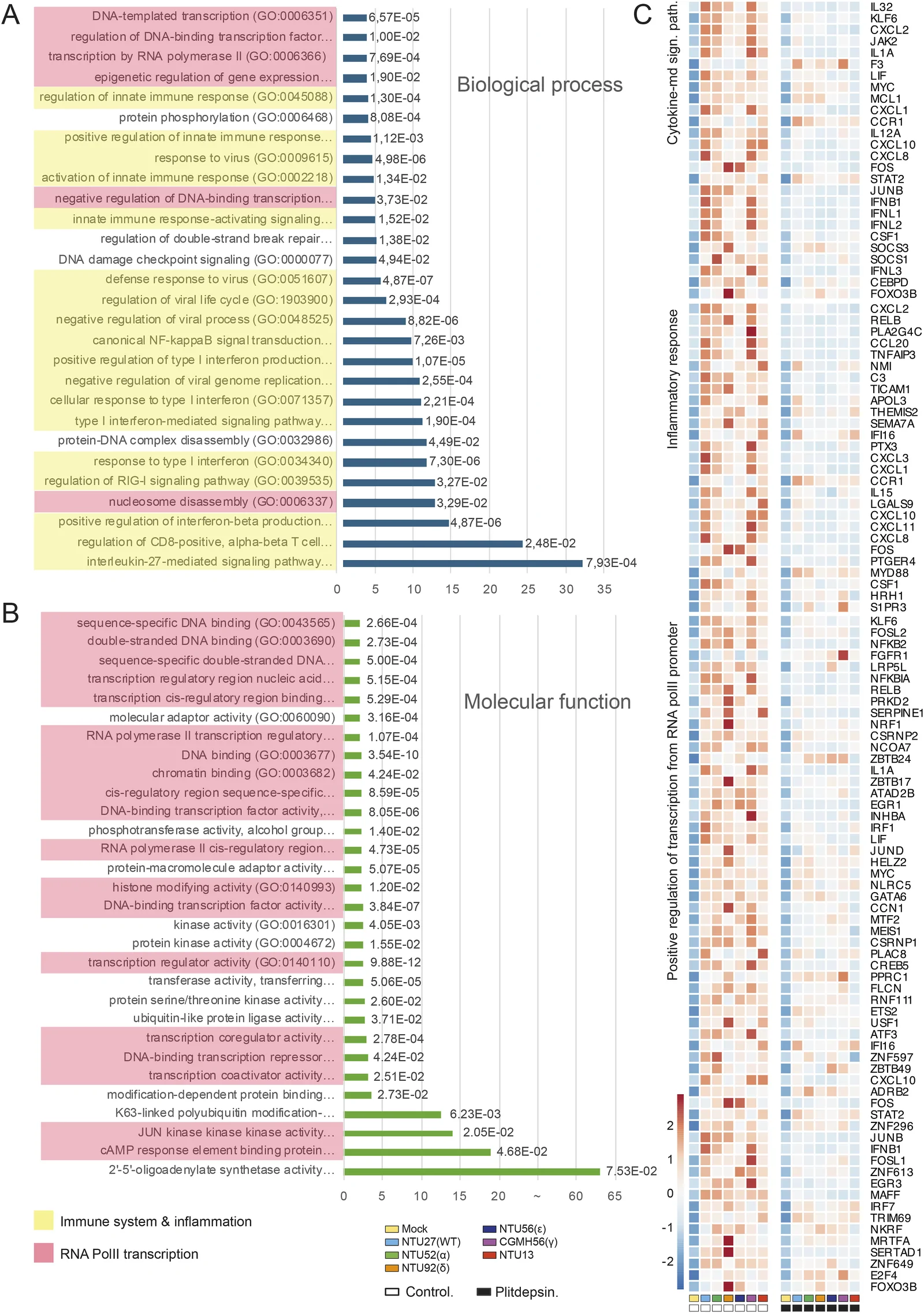
Genome ontology of infection upregulated genes. Gene Ontology–term enrichment analysis of the 337 commonly up-regulated genes (from Fig 1A) in Calu-3 cells infected with SARS-CoV-2 variants. Enriched Gene Ontology terms are shown for Biological Process (A) and Molecular Function (B) highlighting pathways associated with RNA Pol II transcription in red and Immune System & Inflammation in yellow. **(C)** Heatmaps showing the expression of up-regulated genes from the six SARS-CoV-2 variant infection (colored squares, bottom), before (white squares) and after (black squares) treatment with plitidepsin, corresponding to the cytokine-mediated signaling pathway, inflammatory response, and positive regulation of RNA Pol II promoter (indicated on the left of the panels).

Gene Ontology analysis of the main deregulated genes (Fig 1A) revealed significant enrichment in pathways related to RNA Pol II transcription, cytokine activity, and inflammatory response (Fig S3A–C).

Analysis of gene expression confirmed that SARS-CoV-2 infection strongly impacts Pol II transcription and immune pathways and that plitidepsin specifically inhibits these effects. Indeed, infection-up-regulated DNA-binding proteins such as the IRFs (IRF1, IRF8) and IEGs (JUN, FOS, NFκB, CREB) (Fig 1E), as well as cytokine genes (IL6, IFNβ) (Fig 1F), are restored by plitidepsin.

Overall, SARS-CoV-2 variants trigger distinct yet overlapping transcriptional programs marked by Pol II activation and antiviral inflammatory responses, which plitidepsin efficiently counteracts in a dose-dependent manner, partially restoring host gene expression across multiple variants.

### The viral N protein drives host gene expression

Because the N protein from transmissible gastroenteritis coronavirus has been shown to interact with eEF1A (Zhang et al, 2014), we performed immunoprecipitation experiments to investigate whether a similar interaction occurs with the N protein of SARS-CoV-2. Calu-3 cells were transfected with plasmids expressing WT N (N-WT, NTU27), N-R203K (present in most variants), N-D377Y (NTU92, δ), or a deletion mutant lacking amino acids 355–363 (N-del), the putative interaction domain.

**Figure 2.**
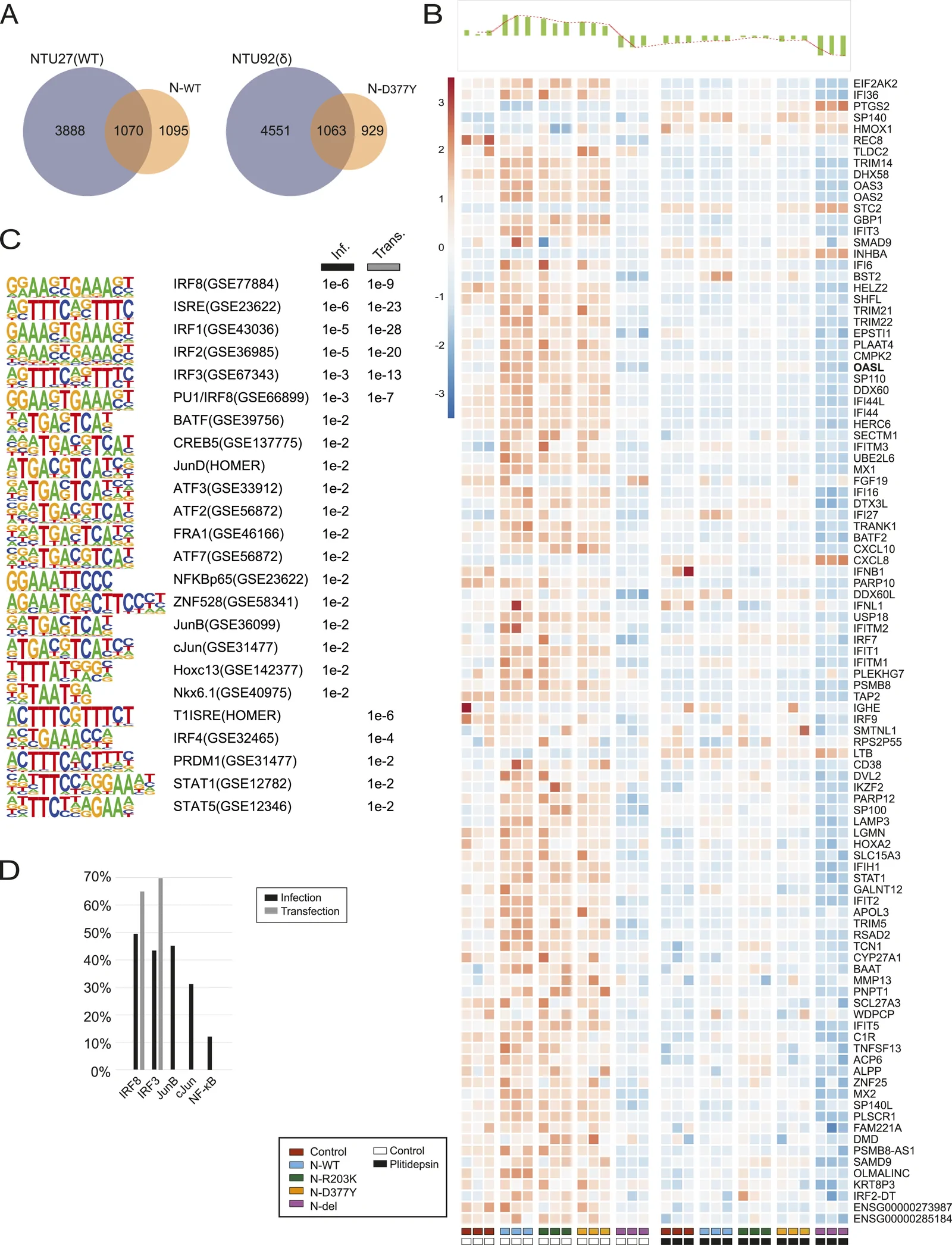
Transfection experiments with the viral N protein. **(A)** Overlap of dysregulated genes in Calu-3 cells. The left panel shows the overlap between DEGs after infection with NTU27(WT) variant and N-WT transfection, whereas the right panel shows the overlap between genes dysregulated after NTU92(δ) infection and N-D377Y transfection. **(B)** Heatmap of RNA-seq expression profiles from Calu-3 cells transfected with the indicated viral N constructs (N-WT, N-R203K, N-D377Y, and N-del; colored squares, bottom), before (white squares) and after (black squares) treatment with 20 nM plitidepsin. The moving average of RNA expression genes is shown at the top. **(C)** DNA-binding motif enrichment identified in the promoters of all the clusters of the 337 up-regulated genes in infected Calu-3 cells (Fig 1A) and the 100 genes in transfected Calu-3 cells (Fig 2B). Enrichment *P*-values are shown on the right side of the panel. **(D)** Percentage of up-regulated genes containing each of the identified motifs for IRF8, IRF3, JunB, c-Jun, or NFκΒ transcription factors within the cluster of 337 genes in infected Calu-3 cells (Fig 1C) and 100 genes in transfected Calu-3 cells (Fig 2B).

After transfection, bulk RNA-seq revealed that many up-regulated genes upon N-WT and N-D377Y transfection overlapped those induced by NTU27(WT) or NTU92(δ) infection, respectively (Fig 2A). Overall, N-WT, N-R203K, or N-D377Y protein transfection induced the overexpression of a large set of genes that cannot be induced by N-del and can be reverted by plitidepsin (Fig 2B). Up-regulated gene promoters from SARS-CoV-2 infection were significantly enriched for DNA-binding motifs recognized by IRFs and IEGs (Fig 2C, black). However, in cells transiently expressing the N-WT protein, induced gene promoters were mainly associated with IRF, whereas IEGs were less represented (Fig 2C, gray). Nonetheless, both infection and N transfection–induced genes are mainly enriched with IRF3 and IRF8 motifs, known for their role in immune response (Fig 2D). The aforementioned difference may also reflect the earlier and transient expression kinetics of IEGs, which might not be captured under our experimental conditions. Notably, IRF regulatory elements were more enriched in N-transfected cells than in infected cells, supporting a specific contribution of the N protein to IRF-driven transcriptional responses during SARS-CoV-2 infection.

### The N protein is recruited at the promoter of activated genes

**Figure S4.**
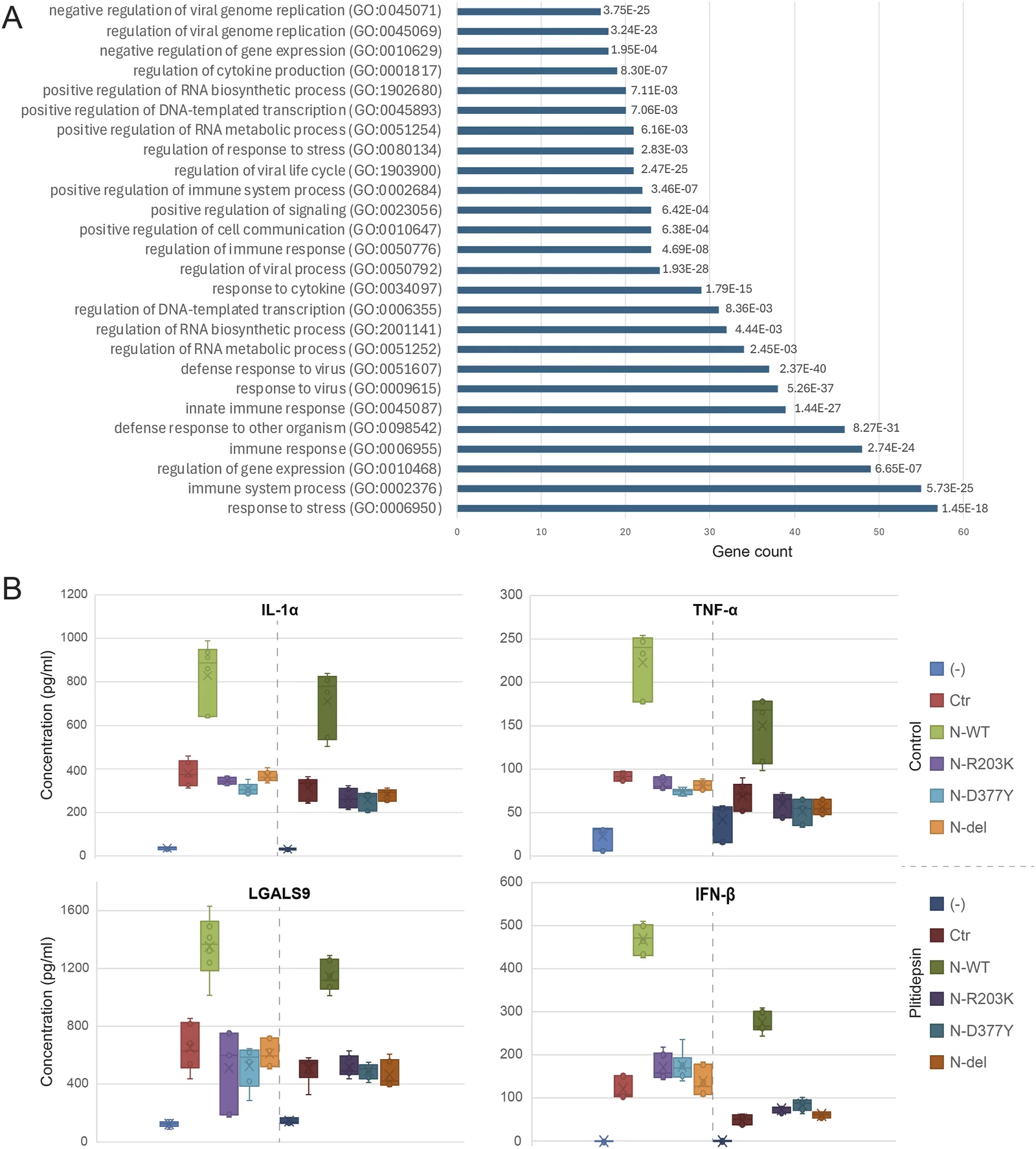
Nucleocapsid effect on immune system. **(A)** Gene Ontology–term enrichment analysis of the up-regulated genes (from Fig 2B) in Calu-3 cells transfected with the SARS-CoV-2 N-WT protein. Enriched Gene Ontology terms are shown for Biological Process. **(B)** ELISA quantification of IL-1α, TNF-α, LGALS9, and IFN-β was performed by multiplex immunoassay on medium supernatant from cells after transfection with WT or mutant viral N proteins, with or without plitidepsin treatment (20 nM).

Gene Ontology analysis of genes up-regulated by N transfection revealed significant enrichment in pathways related to RNA Pol II transcription, cytokine activity, and inflammatory response (Fig S4A). Consistently, N-WT transfection increased the secretion of cytokines and related factors, including IL-1α, TNF-α, LGALS9, and IFN-β, as measured by ELISA in culture supernatants (Fig S4B), and as previously noted (Edalat et al, 2024).

**Figure S5.**
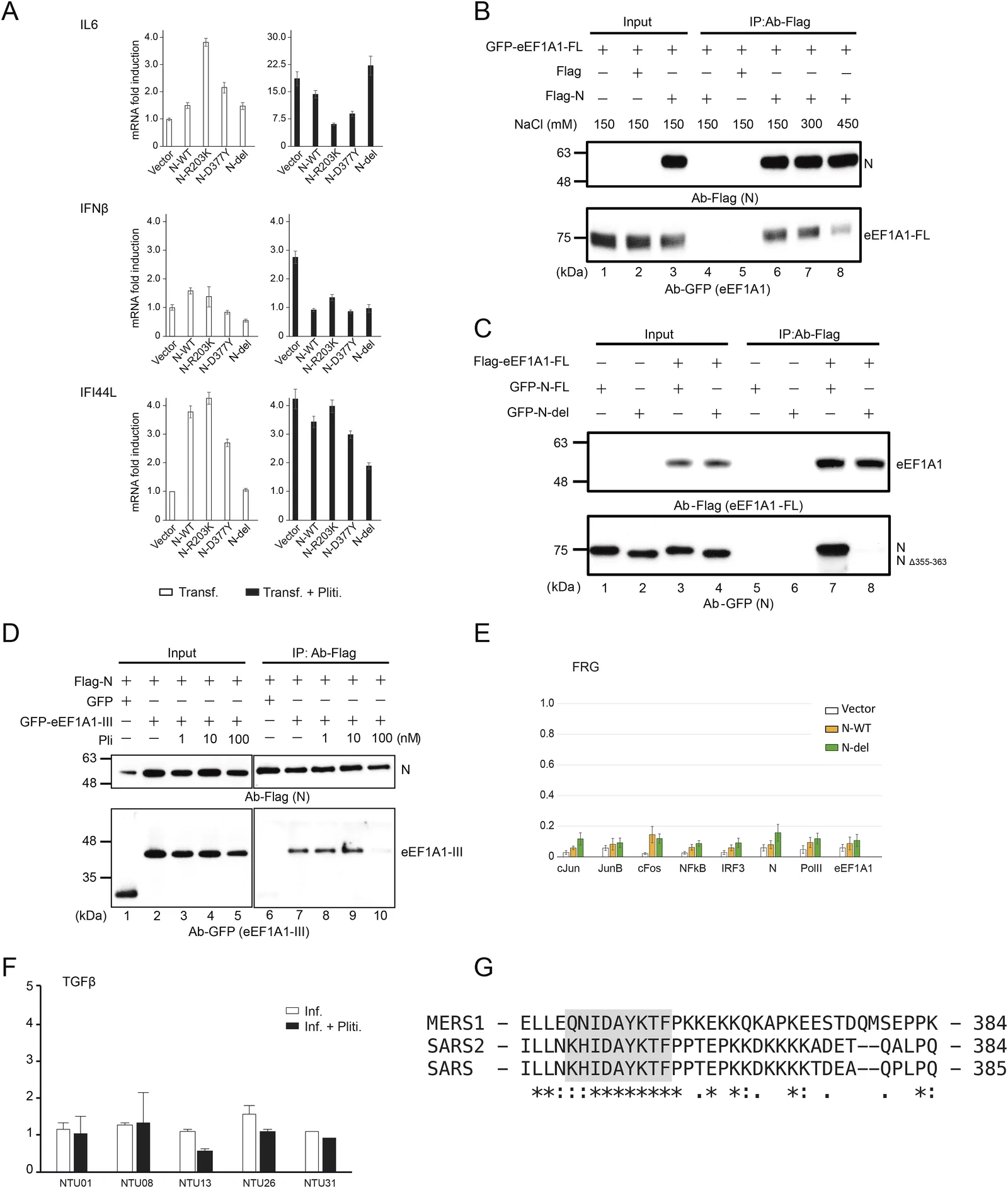
Interaction of eEF1A1 with Nucleocapsid. **(A)** Expression level of IL-6, IFN-β, and IFI44L in Calu-3 cells transfected by vectors expressing viral N-WT, N-R203K, N-D377Y, and N-del with or without 20 nM plitidepsin treatment. RNA extracts were analyzed by RT–qPCR. Each series was monitored in triplicates. Standard deviations are depicted. **(B)** HEK293T cells were cotransfected with Flag-eEF1A1 and GFP-N. Anti-Flag immunoprecipitations from cell lysates were performed, followed by different NaCl concentration washes, and analyzed by immunoblotting using anti-Flag and anti-GFP antibodies. **(C)** HEK293T cells were cotransfected with Flag-eEF1A1 and GFP-N or GFP-N-del. Anti-Flag immunoprecipitations from cell lysates were performed and analyzed by immunoblotting using anti-Flag and anti-GFP antibodies. **(D)** HEK293T cells were cotransfected with Flag-N and either GFP or GFP-eEF1A-III for 24 h and treated with increasing (1–100 nM) concentrations of plitidepsin as indicated. Cell protein extracts were submitted to immunoprecipitation with anti-Flag antibodies and analyzed by immunoblotting with anti-Flag and anti-GFP antibodies. **(E)** Presence of c-Jun, JunB, c-Fos, NFκB, IRF3, N, Pol II, and eEF1A1 was assessed at the promoter of the nonactivated *FGR* region upon transfection of control vector (open square)–, N-WT (yellow square)–, or N-del (green square)–transfected Calu-3. Values represent the percentage of the input. **(F)** Expression level of *TGFβ* in Calu-3 cells infected by various NTU variants treated by 10 nM plitidepsin. It shows the results of three independent experiments. All gene expression levels were normalized to the endogenous internal control gene GAPDH, and the relative expression levels of genes examined in the uninfected Calu-3 cells were defined as 1.0. **(G)** Comparison of SARS-CoV-2, SARS-CoV-1, and MERS-CoV N protein sequences involved in eEF1A1 interaction. Amino acid sequence alignment highlights the regions implicated in interaction with the host translation elongation factor eEF1A1. Conserved amino acid residues are indicated, and residues or regions associated with eEF1A1 binding are highlighted.

**Figure 3.**
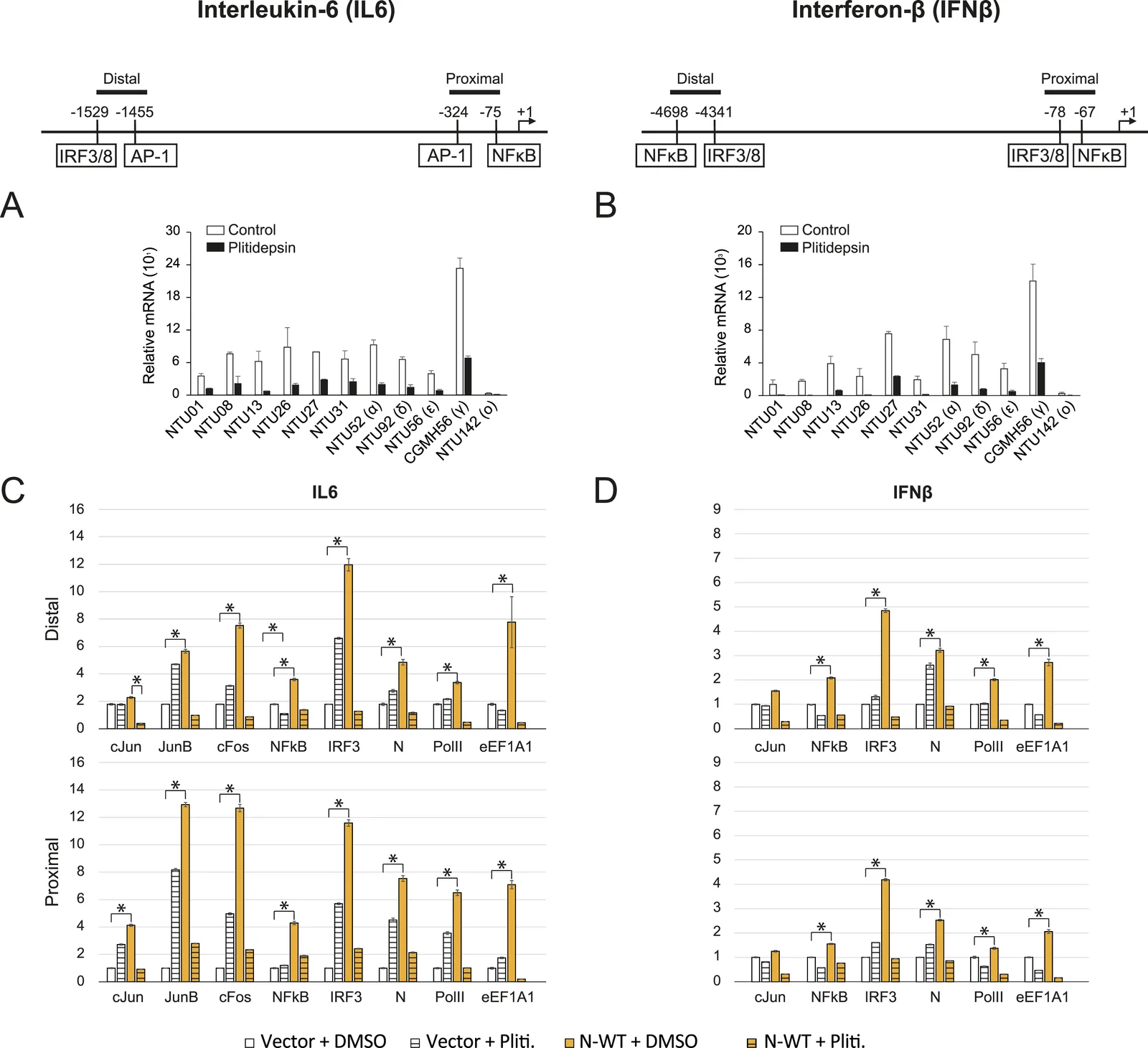
Recruitment of the transcription machinery on the *IL6* and *IFNβ* promoters. Schematic representation of the *IL6* and *IFNβ* promoter regions, showing the locations of IRF3/8, AP-1 (Jun/Fos), and NFκB-binding sites within the distal enhancer and proximal promoter. The +1 position indicates the transcription start site. Expression level of *IL6* (A) and *IFNβ* (B) in Calu-3 cells infected by various NTU variants with or without 20 nM plitidepsin treatment. RNA extracts were analyzed by RT–qPCR. Each series was monitored in triplicates. All gene expression levels were normalized to endogenous internal control gene GAPDH, and the relative expression levels of genes examined in the uninfected Calu-3 cells were defined as 1.0. ChIP-qPCR analysis of transcription factor and protein occupancy at the *IL6* and *IFNβ* loci. The presence of c-Jun, JunB, c-Fos, NFκB, IRF3, N, Pol II, and eEF1A1 was assessed at the distal (upper panels) and proximal (lower panels) regions of the *IL6* (C) and *IFNβ* (D) promoters, respectively, in nontransfected and N-WT transfected cells, with or without plitidepsin treatment, as indicated below each panel. Values represent the percentage of the input. Standard deviation and statistical analyses are depicted (*: *t* test *P*-value 0.05 and Bonferroni correction m = 6). . Source data are available for this figure.

Based on these results, we focused on IL-6 and IFN-β, two key immune mediators. RT–qPCR analysis showed significant up-regulation of both genes in cells transfected with N-WT, N-R203K, N-377Y, or N-del (Fig S5A), closely mirroring the transcriptional patterns observed in cells infected with NTU viral variants (Fig 3A and B). Importantly, treatment with plitidepsin markedly prevents the overexpression of the cytokine genes in N-transfected Calu-3 cells.

Both *IL-6* and *IFN-β* loci contain multiple regulatory elements recognized by IRF3, NFκB, and AP-1(Fos/Jun transcription factors) as depicted on the top of Fig 3. To investigate the mechanism of activation, we performed ChIP-qPCR in Calu-3 cells transfected with the N-WT expression vector or control vector, with or without plitidepsin. Recruitment of transcription factors was analyzed at distal enhancer and proximal promoter regions of *IL-6* and *IFN-β*. At the *IL6* locus, ChIP-qPCR revealed strong enrichment of IRF3, JunB, c-Fos, and Pol II at both distal (−1529 bp) and proximal (−324 bp) regulatory regions (Fig 3C), consistent with enhancer–promoter looping (Le May et al, 2012). In contrast, NFκB and c-Jun showed weaker enrichment. Notably, the viral N protein was detected at the *IL6* promoter and eEF1A1 was also associated with this transcriptional complex. N expression markedly enhanced recruitment of eEF1A1 and the transcriptional machinery. No such recruitment was observed in vector/control-transfected cells, indicating that N promotes assembly of a host transactivation complex. A similar pattern of recruitment was observed at the −4,678-bp enhancer and the −78-bp promoter regions of *IFNβ*, where IRF3 and NFκB were enriched together with Pol II, N, and EF1A1 (Fig 3D). Importantly, treatment of N-transfected cells with plitidepsin abolished recruitment of transcriptional activators (IRF3, AP1, NFκΒ), as well as Pol II, N, and eEF1A1, indicating disruption of the transcriptional complex at these promoter regions.

Together, these results demonstrate that the SARS-CoV-2 N protein is actively recruited to the promoters of key inflammatory genes, where it facilitates the assembly of an eEF1A1-Pol II transcriptional complex, contributing to the transcriptional reprogramming observed during infection (Hasan et al, 2023).

### Biochemical dissection of eEF1A1-N protein binding

**Figure 4.**
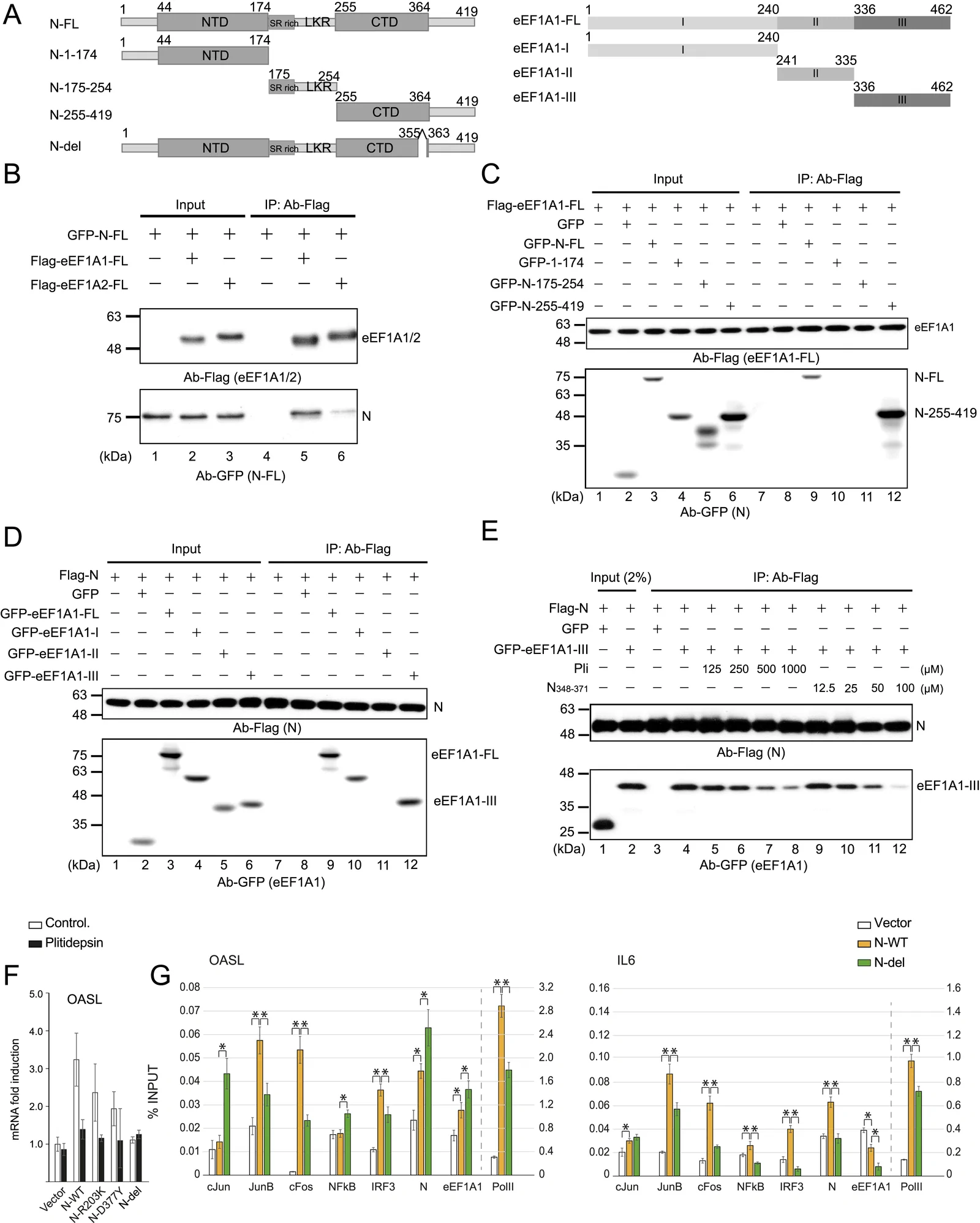
Interaction between SARS-CoV-2 N protein and cellular eEF1A translational factors. **(A)** Schematic representation of plasmid constructs expressing GFP- and Flag-tagged full-length (N-FL) or truncated (N-1-174, N-175-254, N255-419, and N-del), and eEF1A1 (FL) and eEF1A1-I, eEF1A1-II, and eEF1A1-III domains. **(B)** HEK293T cells were cotransfected with GFP-N and either Flag-eEF1A1 or -eEF1A2 for 24 h. Anti-Flag immunoprecipitations from cell lysates were analyzed by immunoblotting using anti-Flag and anti-GFP antibodies. **(C)** Anti-Flag immunoprecipitated fractions from HEK293T cell lysates cotransfected with Flag-eEF1A1-FL and either GFP, GFP-N, GFP-N-1-174, GFP-N-175-254, or GFP-N-255-419 for 24 h were analyzed by immunoblotting with anti-Flag and anti-GFP antibodies. **(D)** HEK293T cells were cotransfected with Flag-N and either GFP-eEF1A1-I, eEF1A1-II, or eEF1A1-III (as indicated panel A) for 24 h and analyzed by immunoblotting with anti-Flag and anti-GFP antibodies. **(E)** HEK293T cell extracts previously cotransfected with Flag-N and eEF1A1-III domain were incubated with increasing amounts of either plitidepsin or N-pep (348–371), during 1 h at 4°C, and further immunoprecipitated with Flag antibodies. The immunoprecipitated fractions were then analyzed by SDS–PAGE and immunoblotting using anti-Flag and anti-GFP antibodies as described. All immunoprecipitations were performed in triplicate. **(F)** Expression level of *OASL* in Calu-3 cells transfected with vectors encompassing viral N-WT, N-R203K, N-D377Y, or N-del, treated or not by plitidepsin (20 nM). RNA extracts were analyzed by RT–qPCR. Standard deviation was calculated over three independent experiments. **(G)** ChIP-qPCR experiments monitoring the presence of c-Jun, JunB, c-Fos, NFκB, IRF3, N, Pol II, and eEF1A1 at *OASL* (left panel) and *IL-6* (right panel) of control vector (open square)–, N-WT (yellow square)–, or N-del (green square)–transfected Calu-3 cells as indicated at the bottom of each panel. qPCR was performed in triplicates; values represent the percentage of the input. Standard deviation and statistical analyses are depicted (*: *t* test *P*-value 0.05 and Bonferroni correction m = 3). Source data are available for this figure.

Having observed the corecruitment of eEF1A1 with the viral N protein at cytokine gene promoters, we next examined the molecular basis of this interaction. eEF1A exists as two isoforms with closely related structures but distinct expression patterns: eEF1A1 is ubiquitously expressed, whereas eEF1A2 is mainly restricted to brain, heart, and skeletal muscle (Mills & Gago, 2021). To confirm the predominant role of eEF1A1 over eEF1A2, HEK293T cells were cotransfected with Flag-tagged eEF1A1 or eEF1A2 together with GFP-tagged full-length N (Fig 4A). Immunoprecipitation assays revealed a markedly stronger association of N-FL with eEF1A1 than with eEF1A2 (Wang et al, 2025; Fig 4B), which remained stable under high-salt conditions (300 mM NaCl), indicating robust binding (Fig S5B).

Mapping experiments using N truncation mutants showed that eEF1A1 interacts with the C-terminal region of N (aa 255–419) (Fig 4C). Deletion of residues 355–363 (N-del) almost completely abolished this interaction, identifying this motif as critical for binding (Fig S5C). Domain mapping further showed that both domain I and domain III of eEF1A1 contribute to N binding, with domain III mediating the strongest interaction (Fig 4D).

To characterize the interaction interface between eEF1A1 and the N protein, we employed a synthetic peptide corresponding to residues 348–371 of the N protein’s C-terminal domain (N348-371) as a competitive inhibitor. Cellular lysates co-expressing Flag-tagged full-length N protein and GFP-tagged eEF1A1 domain III were incubated with increasing concentrations of the N348-371 peptide (12.5–100 μM) (Fig 4E, lanes 9–12). The N348-371 peptide efficiently competed with the N/eEF1A1 interaction in vitro. Similarly, plitidepsin disrupted the interaction in lysates at micromolar concentrations (Fig 4E, lanes 4–8), whereas in cells, effective inhibition was observed at nanomolar concentrations (Fig S5D).

To assess the functional relevance of this interaction, we examined *OASL*, an interferon-induced gene essential in innate antiviral defense (Rivera-Cardona et al, 2026), and up-regulated after SARS-COV-2 infection (Fig 1A and C) or N protein transfection (Fig 2B). RT–qPCR analysis revealed strong induction of *OASL* in cells transfected with either N-WT or N mutant constructs, whereas the N-del mutant failed to induce *OASL* up-regulation (Fig 4F). Consistently, plitidepsin treatment reversed N-induced *OASL* expression.

ChIP analysis revealed that N-WT promotes the recruitment of JunB and c-Fos (AP-1), IRF3, N, eEF1A1, and Pol II to the *OASL* promoter (Fig 4G, left panel) and *IL-6* promoter (Fig 4G, right panel). In contrast, the N-del mutant, which lacks the eEF1A1-binding domain, showed significantly reduced recruitment of Pol II and IRF3, AP-1 transcriptional activators, thereby explaining the low *OASL* expression observed upon N-del transfection (Fig 4F). Similarly, Pol II recruitment to the *IL6* promoter was markedly impaired in Calu-3 cells transfected with the N-del protein, along with the reduced presence of eEF1A1, IRF3, and AP-1, consistent with the decreased IL-6 expression levels. Interestingly, N-del and eEF1A1 still accumulated, even more strongly at the *OASL* promoter (Fig 4G). This suggests potential additional interactions between eEF1A1 and the N-terminal region in some cases, mediated by other transcriptional components (Fig 4D), potentially leading to the formation of an inactive transcription initiation complex.

As a control, N, eEF1A1, JunB, and Pol II were not recruited to the promoter of the nonactivated FGR gene (Fig S5E). Similarly, *TGF-β*, which lacks IRF and IEG responsive elements, was not up-regulated upon NTU variant infection (Fig S5F).

Together, these results identify a functional interaction between the SARS-CoV-2 N protein and eEF1A1, mediated primarily by the N protein C-terminal region. Disruption of this interface, either genetically or pharmacologically, impairs recruitment of the transcriptional machinery and blocks N-driven host transcriptional reprogramming, highlighting the N-eEF1A1 axis as a potential antiviral target.

## Discussion

In this study, we identify a conserved mechanism by which SARS-CoV-2 reprograms host gene expression across genetically diverse viral variants. Comparative transcriptomic analysis of Calu-3 cells infected with multiple SARS-CoV-2 lineages including Alpha, Gamma, Delta, Epsilon, and Omicron revealed a highly conserved host transcriptional response despite substantial viral genetic divergence. Infection consistently dysregulated thousands of host genes and triggered activation of IRFs, NFκB targets, and IEGs (e.g., JUN, FOS, ATF, CREB), followed by induction of pro-inflammatory cytokines, reflecting the hierarchical organization of the antiviral response.

Mechanistic analyses identify the viral N protein as a central driver of this transcriptional reprogramming. The expression of N alone recapitulated a substantial portion of the transcriptional program induced during infection, including activation of IRF- and IEG-regulated genes. Alterations within the N protein significantly modify host transcriptional responses, consistent with previous reports linking N mutations to changes in viral pathogenicity (Mourier et al, 2022). Notably, the recent Omicron subvariants such as BA.2 and XBB, which contain specific N mutations, induced stronger transcriptional activation than the early Omicron isolate, suggesting continued viral adaptation toward enhanced modulation of host immune pathways. ChIP-qPCR analyses revealed that N promotes recruitment of IRF3, IRF8, Jun, Fos, and Pol II to cytokine gene promoters such as *IL6*, *IFNβ*, and *OASL*, highlighting an unexpected nuclear role for N beyond its canonical function in viral RNA packaging.

A key finding of this work is the identification of a functional interaction between SARS-CoV-2 N protein and the translation elongation factor eEF1A1 at the transcriptional level. Although eEF1A1 has traditionally been viewed as a core component of the translational machinery, our data reveal that it also contributes to transcriptional regulation during SARS-CoV-2 infection. Although this may appear counterintuitive from the perspective of viral replication, we demonstrate that eEF1A1 co-occupies cytokine promoters with IRF and AP-1 transcription factors, suggesting that SARS-CoV-2 co-opts this abundant host factor to facilitate the assembly or stabilization of transcriptional complexes that promote pro-inflammatory gene expression. Consistent with this model, disruption of the N-eEF1A1 interaction either pharmacologically using the eEF1A interactant plitidepsin or with a competitive peptide abolished transcriptional complex formation, restored normal host gene expression, and suppressed viral replication across multiple variants. These observations also provide a molecular explanation for the reduced transcription of early-response genes such as NFκB, a key mediator of the inflammatory cascade, as well as for the decreased levels of pro-inflammatory cytokines, including IL-6, IFN-β, TNF-α, observed both in the nonproductive SARS-CoV-2 infections in vitro and in the lungs of SARS-CoV-2–infected mice treated with plitidepsin (Losada et al, 2024). Together with recent reports that the N protein modulates cGAS-IRF3 signaling (Gutmann et al, 2025), our data support a broader model in which SARS-CoV-2 exploits multifunctional host proteins to coordinate transcriptional rewiring of the infected cells.

Viruses employ diverse strategies to manipulate host gene expression. For example, Rift Valley fever virus targets transcription factor TFIIH to suppress host transcription (Le May et al, 2004), whereas Epstein-Barr virus redirects transcriptional machinery to newly activated promoters (Ungerleider et al, 2024), and other viruses rely on epigenetic modification of promoter elements (Locatelli & Faure-Dupuy, 2023). In contrast, our findings indicate that SARS-CoV-2 repurposes a component of the translational machinery to assemble transcriptional complexes that activate host inflammatory programs.

Interactions between eEF1A and viral proteins have been reported for several viruses, including HIV-1 (Cimarelli & Luban, 1999), hepatitis B virus (Lin et al, 2012), and coronaviruses (Zhu et al, 2021), highlighting eEF1A as a conserved host factor frequently co-opted during infection. However, these studies have primarily linked eEF1A to viral translation or RNA replication. Our results extend this paradigm by demonstrating that the SARS-CoV-2 N protein recruits eEF1A1 to cytokine gene promoters together with transcription factors and Pol II, thereby revealing an unexpected role for eEF1A1 in transcriptional regulation.

Because the N protein is highly conserved among SARS-CoV-1, MERS-CoV, and SARS-CoV-2 (Fig S5G), the N-eEF1A1 interaction represents a compelling pan-coronavirus target. Supporting this view, evidence from infected mouse models, early clinical studies, and emerging Phase III data indicates that plitidepsin effectively limits SARS-CoV-2 replication and inflammation (Varona et al, 2023; Jimeno et al, 2025).

**Figure S6.**
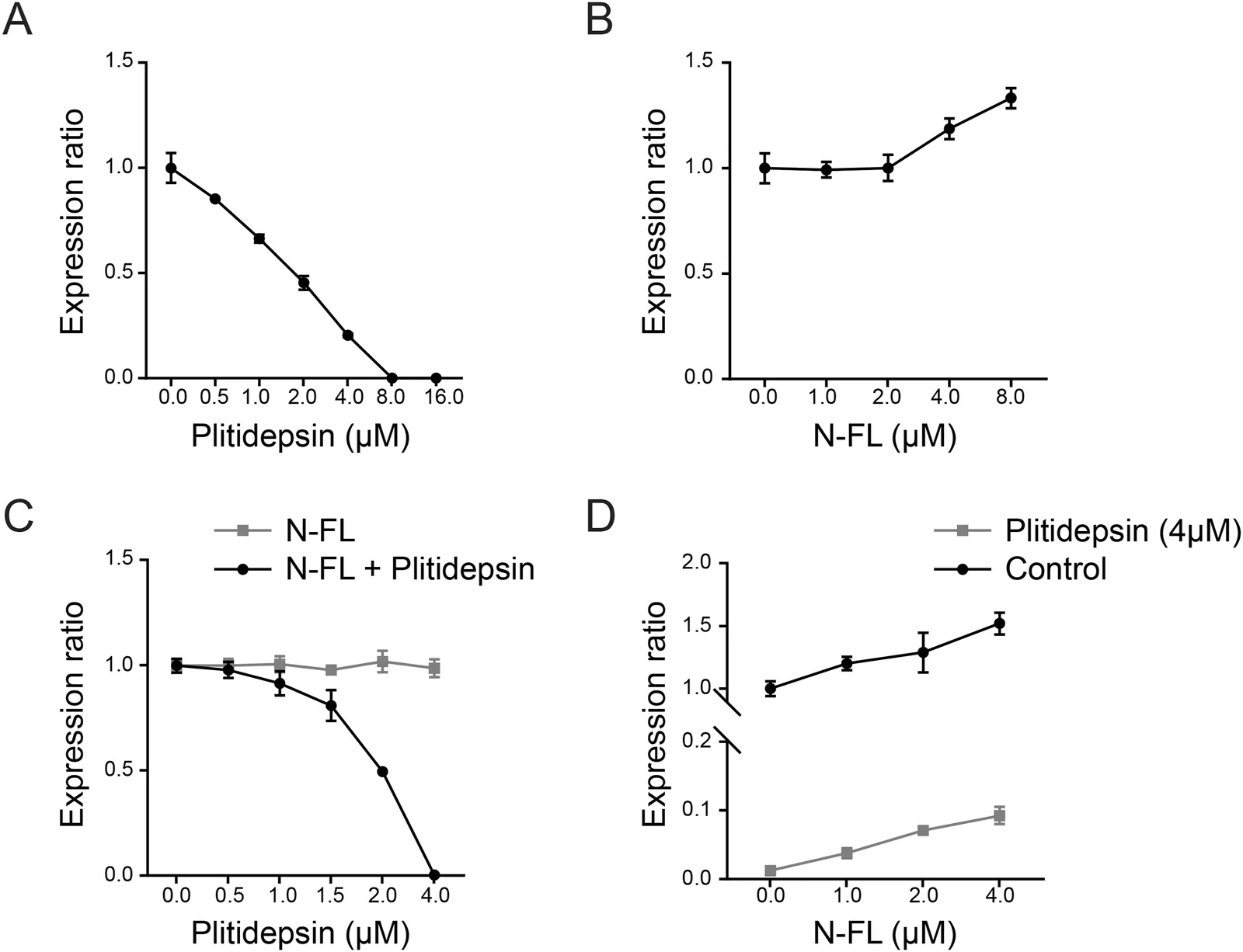
Effect of Nucleocapsid and Plitidepsin on protein translation. **(A)** Luciferase production by Rabbit Reticulocyte Lysate and an increasing concentration of Plitidepsin. **(B)** Luciferase production by Rabbit Reticulocyte Lysate in presence of increasing concentration of the Nucleocapsid protein from SARS-CoV-2, from 0 µM up to 8 µM. **(C)** Luciferase production by Rabbit Reticulocyte Lysate with a fix concentration of the Nucleocapsid protein of 8 µM and an increasing concentration of Plitidepsin up to 4 µM. **(D) **Luciferase production by Rabbit Reticulocyte Lysate with a fix concentration of of Plitidepsin at 4 µM and an increasing concentration of Nucleocapsid protein of 8 µM.

Collectively, these findings define a host–virus regulatory axis in which the N-eEF1A1 interaction operates at the transcriptional level to reprogram host gene expression. However, one cannot exclude that perturbation of eEF1A1 function (by the viral N) at the translational level may interfere with the protein synthesis programs required for efficient viral replication (Fig S6). Nevertheless, this transcription reprogramming induced by SARS-CoV-2 may provide a unifying explanation for the diverse cellular alterations observed during infection, including changes in lysosomal pathways, cytoskeleton dynamics and organization, and viral genome replication ultimately reshaping cellular physiology to favor efficient virion production and contributing to SARS-CoV-2 pathogenesis. Importantly, the N-eEF1A1 interaction may represent a therapeutically relevant host–virus interface that operates at both the transcriptional and translational levels. By targeting the host factor eEF1A1, plitidepsin may disrupt these virus-induced cellular programs rather than directly targeting a viral enzyme, potentially limiting both viral replication and the associated dysregulation of host gene expression. This mechanism differs from that of direct-acting antivirals such as nirmatrelvir/ritonavir, remdesivir, and molnupiravir, which primarily target viral protease or viral RNA synthesis and therefore act directly on processes associated with active viral replication.

Targeting a host dependency such as eEF1A1 could thus offer an interesting therapeutic strategy while limiting possible resistance mechanisms. It brings the potential to interfere with viral replication by mitigating the host transcriptional and translational programs exploited by SARS-CoV-2. Together, these findings position the N-eEF1A1 interaction as a promising target for host-directed antiviral therapy.

## Materials and Methods

### Eukaryotic cell lines and culture medium

Eukaryotic Calu-3 and HEK293T cell lines were originally obtained from ATCC. HEK293T cells were cultured in DMEM containing 10% FBS and 1% penicillin/streptomycin. Calu-3 cells were cultured in DMEM containing 15% FBS, 1% penicillin/streptomycin, 2 mM L-glutamine (GlutaMAX; Gibco). All the cells were cultivated in a 37°C humidified incubator with 5% CO_2_.

### SARS-CoV-2 infection

Calu-3 cells were seeded in 24-well plates (2 × 10^5^ cells/well) and incubated for 4 d. The culture medium was then replaced with serum-free DMEM containing 2 μg/ml TPCK-trypsin and SARS-CoV-2 virus (MOI = 0.1) for a 1-hour infection. Afterward, the supernatant was removed, cells were washed once with PBS, and DMEM (2% FBS, 1% penicillin/streptomycin, 2 mM L-glutamine) was added for a 24-h incubation postinfection. The culture supernatant was collected for viral RNA quantification using RT–qPCR and PFU by the plaque assay.

### Viral load quantification

Viral RNA was extracted from 140 μl of supernatant using QIAamp Viral RNA Extraction Kit (QIAGEN). The extracted RNA was solubilized in 60 μl of nuclease-free water. Viral RNA quantification was performed using One-Step Probe RT–qPCR Kit (TOOLS) and 7500 Real-Time PCR System (Applied Biosystems, Thermo Fisher Scientific). The probe targeting the viral E gene and other primers were purchased from Integrated DNA Technologies. A standard curve for viral RNA was created using an E gene RNA standard with 103, 104, 105, 106, 107, and 108 copies. The Ct value of viral RNA was then converted to copies using the standard curve.

### Plaque reduction assay

Calu-3 cells were seeded in 24-well plates (2 × 10^5^/well) and incubated for 24 h. The cells were infected with 150 PFU/well of SARS-CoV-2 and various concentrations of plitidepsin (serial two-fold dilution up to 20 nM) for 1 h. The supernatant was removed, and the cell monolayer was washed once with 1X PBS buffer, before overlaid with medium containing the indicated concentrations of plitidepsin, 1% methylcellulose, and 2% FBS, followed by an additional incubation for 5–7 d. Cells were fixed with 10% formaldehyde overnight, the overlay medium was removed, and the cells were stained with 0.5% crystal violet. The plaques were manually counted. The percentage of inhibition was calculated as [1 − (VD/VC)] × 100%, where VD and VC represent the virus titer in the presence and absence of the compound, respectively. The minimal concentration of the compound required to reduce 50% of the plaque numbers (EC50) was calculated by regression analysis of the dose–response curves generated from the plaque reduction assays. All experiments involving SARS-CoV-2 virus were performed in the Biosafety Level-3 Laboratory of the National Taiwan University College of Medicine.

### Plasmids and transfection

DNA plasmids encoding tagged-N and tagged-eEF1A1 fusion proteins were constructed with material described in Table S1. The backbone vectors, inserts, restriction enzymes, and primers used to build the different plasmids are listed. All plasmids were transfected into HEK293T or Calu-3 cells for 24 h using Lipofectamine 3000 (Thermo Fisher Scientific), according to the manufacturer’s instructions.

Table S1. Sequence of the primers used for the cloning of eEF1A1 and N in GFP and flag vectors.

### RNA extraction and RT–qPCR assay

Cellular RNA was extracted using Smart RNA Extractor (TOOLS), and cDNA was synthesized using Quant II Fast RT Kit (TOOLS). RT–qPCR was later performed with SYBR Green (MorreBio) and 7500 Real-Time PCR System (Applied Biosystems, Thermo Fisher Scientific). All gene expression levels were normalized to the housekeeping gene GAPDH. All primer sequences are available in Table S2.

Table S2. Sequence of the primers used for qPCR for RNA and ChIP experiments.

### Co-immunoprecipitation

Cell pellets were resuspended in IP buffer (150 mM NaCl, 0.5% Na-deoxycholate, 1% NP-40, 5 mM EDTA, 50 mM Tris–HCl, pH 8.0) containing protease inhibitor (cOmplete Tablets EDTA-free, Roche), then sonicated to lyse cells. The lysate was incubated with Anti-Flag M2 Affinity Gel for 1 hr (4°C), centrifuged at 8,000 rpm (1 min, 5,9*g*), and washed with IP buffer, then resolved by SDS–PAGE and Western blot with indicated antibodies. Plitidepsin and N348-371 peptides were added to protein extract at the indicated concentration and incubated for 2 h and 1 h, respectively, before immunoprecipitation.

### Chromatin immunoprecipitation

After transfection and treatment, Calu-3 cells were cross-linked at room temperature for 15 min with 1% formaldehyde. All subsequent steps were performed at 4°C. Cells were resuspended in swelling buffer to isolate nuclei (Tris–HCl 50 mM, pH 8, EDTA 10 mM, SDS 1%), then centrifuged at 4,000 rpm for 10 min. The nucleus pellet was resuspended in sonication buffer (50 mM Hepes, pH 7.9, 140 mM NaCl, 1 mM EDTA, 1% Triton X-100, 0.1% Na-deoxycholate, 0.1% SDS) and sonicated for 25 min in total on a Q700 QSonica as previously described (Bidon et al, 2018). Samples were immunoprecipitated with antibodies at 4°C overnight, and magnetic protein A/G beads (Upstate) were added and incubated for 4 h. The beads were washed repeatedly with modified sonication buffer with 300 mM NaCl. Protein–DNA complexes were eluted (NaHCO3 0.1 M, SDS 1%) followed by RNase A and proteinase K treatment. Finally, DNA fragments were purified using the QIAquick PCR purification kit (QIAGEN) and analyzed by quantitative PCR. All primer sequences are available in Supplemental Material.

### Antibodies

Immunoprecipitation: anti-Flag M2 Affinity Gel (#A2220-5Ml, Lot #SLCG5835; Sigma-Aldrich), EF1A1 (#11402-1-AP; Proteintech), c-Jun (60A8; Cell Signaling), JunB (C37F9; Cell Signaling), c-Fos (sc52; Santa Cruz), NfkB (D14E12; Cell Signaling), IRF3 (D6I4C; Cell Signaling), Pol II (Rpb1, D8L4Y; Cell Signaling).

Immunoblotting: GFP (#113617, Lot #40555; GeneTex; #2956, Lot #6; Cell Signaling Technology), Flag (#T503-2, Lot #5117; Signalway Antibody; #14793, Lot #7; Cell Signaling Technology), EF1A1 (#11402-1-AP; Proteintech; #sc-21758, Lot #C1122; Santa Cruz Biotechnology), EF1A2 (#102326, Lot #42705; GeneTex), SARS-CoV-2 N (#632269, Lot #43951; GeneTex), GAPDH (#100118, Lot #43712; GeneTex).

### Cytokine quantification

Cell culture medium was collected and quick frozen in liquid nitrogen for sending to Inflammation Core Facility of the Institute of Biomedical Sciences, Academia Sinica. After receipt, samples were thawed, mixed thoroughly by vortexing, and centrifuged at 15,000*g* for 5 min to remove particulates. Human IL-1α, TNF-α, IFN-β, and LGALS9 were analyzed without further dilution (1×).

Human IL-1α, TNF-α, IFN-β, and LGALS9 concentrations in cell culture medium samples were quantified using a Luminex-based multiplex immunoassay at the Inflammation Core Facility, Institute of Biomedical Sciences, Academia Sinica, Taiwan, supported by the Academia Sinica Core Facility and Innovative Instrument Project (AS-CFII-113-A9). Antibody-conjugated magnetic beads were incubated with the appropriately prepared samples in 96-well microtiter plates. A total of 50 μl of each sample, standard, and quality-control preparation was added to the corresponding wells and incubated with premixed microbeads for 2 h on an orbital plate shaker at room temperature. The plates were washed three times with assay wash buffer, followed by the addition of 40 μl of biotinylated detection antibody to each well. After incubation for 1 h at room temperature on the plate shaker, the wells were washed, and 50 μl of streptavidin-phycoerythrin solution was added. The plates were incubated for an additional 30 min at room temperature on the plate shaker and protected from direct light. Fluorescence signals were acquired using a Luminex multiplex analyzer, and analyte concentrations were calculated from the corresponding standard curves. All incubation and signal-detection steps were performed at room temperature and protected from light.

Antibody-conjugated magnetic beads and biotinylated detection antibodies were prepared and used at the validated working conditions established for the corresponding human multiplex panel. Streptavidin-phycoerythrin was used as the fluorescent reporter.

### RNA-seq, bioinformatics, and statistics analysis

Cellular RNA was extracted using Smart RNA Extractor (TOOLS). The cDNA synthesis and the multiplex cDNA library were prepared using Universal V8 RNA-seq Library Prep Kit for Illumina (VAHTS) and sequenced on the Illumina NovaSeq 6000 with 150-bp paired-end read. After quality filtering and adapter trimming by Cutadapt (v1.9.1), the reads were mapped to the ENSEMBL GRCh38 human reference genome assembly by Hisat2 (v2.0.1) with default parameters (Kim et al, 2015). The normalization and removal of low-count features were performed by the proportion test and by the median of ratios method, respectively, using the NOISeq package (v2.38.0) 49 in R (v4.1.3) (R Core Team, 2020). Genes with very low expression (CPM < 1 in all samples) were excluded before analysis.

Comparative analysis of DEGs was performed pairwisely between the samples using the *noiseq* function in the NOISeq package with default parameters (Tarazona et al, 2015). As per NOISeq recommendation, q = 90 and q = 80 were used as the probability of differential expression for the simplicate (infection) and triplicate (transfection) experiment, respectively. The down-regulated and up-regulated DEGs were tested for Gene Ontology–term enrichment analysis using https://geneontology.org/. The plots associated with RNA-seq were generated using different R packages, including pheatmap, venndiagram, ggplot.

### Statistical analysis

For ChIP-qPCR, samples immunoprecipitated with the same antibody were tested for statistical divergence by analysis of variance, with the corresponding number of factors and Bonferroni correction. When meaningful, standard pairwise *t* tests were performed to determine statistically significant differences.

## Supplementary Material

Reviewer comments

## Data Availability

Raw sequencing data of RNA-seq were deposited in the NCBI SRA under BioProject ID PRJNA976075, PRJNA1380047, and PRJNA1378744.
