# Supplementary material for: SARS-CoV-2 nucleocapsid protein hijacks host transcription by targeting eEF1A1
Source: Life Sci Alliance. 2026 Oct 14;9(12):e202603751. doi: 10.26508/lsa.202603751 (PMC13669674; doi:10.26508/lsa.202603751)
Supplement: Supplementary file 1 [file LSA-2026-03751_review_history.pdf]

SARS-CoV-2 Nucleocapsid protein hijack host transcription by targeting eEF1A1.

Baptiste Bidon, Yu-Lin Lai, Tai-Ling Chao, Gawa Chen, Yee-Shin Lee, Hsiang-Hung Huang, Jia-Syuan Chen, Alejandro Gonzalez Iosada, Sui-Yuan Chang, Jean-Marc Egly, and Tsai-Kun Li
DOI: https://doi.org/10.26508/lsa.202603751

Corresponding author(s): Jean-Marc Egly, Institut de génétique et de biologie moléculaire et cellulaire

Review Timeline:

Submission Date:	2026-04-29
Editorial Decision:	2026-07-06
Revision Received:	2026-08-20
Editorial Decision:	2026-09-14
Revision Received:	2026-09-24
Accepted:	2026-09-24

Scientific Editor: Tim Fessenden

Transaction Report:

(Note: With the exception of the correction of typographical or spelling errors that could be a source of ambiguity, letters and reports are not edited. The original formatting of letters and referee reports may not be reflected in this compilation.)

July 6, 2026

Re: Life Science Alliance manuscript #LSA-2026-03751-T

Jean-Marc Egly
IGBMC
France

Dear Dr. Egly,

Thank you for submitting your manuscript entitled "Hijacking Host Transcription by targeting eEF1A1: A Novel Function of N, the SARS-CoV-2 Nucleocapsid Protein." to Life Science Alliance. The manuscript was assessed by two expert reviewers, whose comments are appended to this letter. We invite you to submit a revised manuscript addressing their main concerns.

As you will see, both reviewers found the results on eEF1A1-N protein interaction intriguing and valuable to the field. Reviewer 2 felt that the partial inhibition of eEF1A1 at the doses of plitidepsin used here should be more directly discussed, and dose-dependent effects of plitidepsin should be shown explored (point 2). This reviewer also requested sequence comparisons with MERS and SARS-CoV N protein (point 3). Both reviewers made important suggestions to better position this work relative to prior literature. Reviewer 1 also requested statistical analysis for key results. While the request to recapitulate results with SARS-CoV-2 infected cells would strengthen this work (Reviewer 2, point 3), this point is optional in a revision.

I would be happy to discuss the revision in more detail via email or phone/videoconferencing. Please let me know which option you prefer, if any.

To upload the revised version of your manuscript, please log in to your account: https://lsa.msubmit.net/cgi-bin/main.plex

You will be guided to complete the submission of your revised manuscript and to fill in all necessary information. Please get in touch in case you do not know or remember your login name.

The typical timeframe for revisions is three months. Please note that papers are generally considered through only one revision cycle, so strong support from the referees on the revised version is needed for acceptance.

While you are revising your manuscript, please also attend to the below editorial points to help expedite the publication of your manuscript. Please direct any editorial questions to the journal office. When submitting the revision, please include a letter addressing the reviewers' comments point by point.

Thank you for this interesting contribution to Life Science Alliance. We hope that the comments below will prove constructive as your work progresses, and we are looking forward to receiving your revised manuscript.

Sincerely,

Tim Fessenden
Scientific Editor
Life Science Alliance

A. THESE ITEMS ARE REQUIRED FOR REVISIONS

-- A letter addressing the reviewers' comments point by point.

-- An editable version of the final text (.DOC or .DOCX) is needed for copyediting (no PDFs).

-- High-resolution figure, supplementary figure and video files uploaded as individual files: See our detailed guidelines for preparing your production-ready images, https://www.life-science-alliance.org/authors

-- Summary blurb (enter in submission system): A short text summarizing in a single sentence the study (max. 200 characters including spaces). This text is used in conjunction with the titles of papers, hence should be informative and complementary to the title and running title. It should describe the context and significance of the findings for a general readership; it should be written in the present tense and refer to the work in the third person. Author names should not be mentioned.

-- By submitting a revision, you attest that you are aware of our payment policies found here: https://www.life-science-alliance.org/copyright-license-fee

B. MANUSCRIPT ORGANIZATION AND FORMATTING:

Full guidelines are available on our Instructions for Authors page, https://www.life-science-alliance.org/authors

We encourage our authors to provide original source data, particularly uncropped/-processed electrophoretic blots and spreadsheets for the main figures of the manuscript. If you would like to add source data, we would welcome one PDF/Excel-file per figure for this information. These files will be linked as supplementary "Source Data" files.

IMPORTANT: It is Life Science Alliance policy that if requested, original data images must be made available. Failure to provide original images upon request will result in unavoidable delays in publication. Please ensure that you have access to all original microscopy and blot data images before submitting your revision.

Reviewer #1 (Comments to the Authors (Required)):

1) The study focuses on the role of eEF1A and its potential as a host-targeted anti-viral using transcriptomics to identify how existing treatments affect viral replication, and how this ties into the broad-spectrum activity of plitidepsin. The principal advancement being how eEF1A-1 in particular interacts with SARS-CoV's N protein, and how this interaction causes transcriptomic dysregulations that lead to the hijacking of the cell's machinery, concluding that plitidepsin works by disrupting this interaction.
2) Antiviral activities of SARS-CoV 2 variants: I don't think this section is particularly relevant for the focus of the rest of the paper. I would suggest keeping the last paragraph and citing the S1 once.
Differential expression profiles induced by the infection of SARS-CoV2 variants: The whole first paragraph belongs in methods, I would move it completely. Volcano plots seem a bit redundant with the use of heatmaps on the same figure; they distract from the overall narrative.
The viral N protein drives host gene expression: 2D seems irrelevant considering what's shown in 2C.
The N protein is recruited at the promoter of activated genes: There's no indication of any statistical analysis in any of these graphs. I would strongly suggest including those results in the graphs, as it'll give the results more robustness.
Biochemical Dissection of eEF1A1-N Protein Binding: The first sentence is duplicated. Again, statistical analysis would be helpful. Extensive methodology details should be in the methods section.
I would suggest adding a different positive control to accompany Plitidepsin, a comparison with other antivirals in this specific context would support your thesis more rigorously.

3) I would suggest rigorous editing of the text; it is repetitive and lacks proper structure. There are multiple spelling, grammar, and punctuation errors, repetitions, and large portions of the results are occupied by explaining the methodology.

Reviewer #2 (Comments to the Authors (Required)):

In this manuscript, Bidon and col. present novel concepts related to the role of the nucleocapsid (N) of SARS-CoV-2, which drives a host transcriptional reprograming via eEF1A that acts in this context as a nuclear factor involved in transcription of pro-inflammatory genes. While the work offers valuable novel insights identifying a conserved mechanism by which SARS-CoV-2 reprograms host gene expression across genetically diverse viral variants, it lacks a better contextualization of prior studies and the mechanism of action exerted by plitidepsin, the drug used to block eEF1A in this paper.

Key points that should be reinforced include:

1. In the introduction, the authors state that "Plitidepsin inhibits the translation machinery and has shown promising activity in clear-cell ovarian cancer 26,27, and against SARS-CoV-2 28. However, the mechanisms underlying its antiviral effects remain poorly understood".

Yet there are several studies that have gain key molecular insights explaining how plitidepsin displays its antiviral effect, that even go beyond inhibiting eEF1A:

*In the seminal study by White et al. (PMID: 33495306) it was already shown that plitidepsin was inhibiting eEF1A, having a much greater impact on subgenomic viral RNA than on genomic RNA, especially when full inhibitory concentrations of the drug were used.

*Later reports efficiently showed how plitidepsin inhibit the replication cycle by inhibiting the formation of double membrane vesicles where subgenomig RNA is formed (PMID: 35231500), affecting different SARS-CoV-2 variants with equal efficacy as shown in this manuscript.

*Recently, it has been shown that plitidepsin effectively blocks cap-dependent and IRES-mediated translation via the inhibition of the elongation factor eEF1A along with the bystander phosphorylation of eF2, which is a key regulator of protein translation initiation that is also blocked by this drug (PMID: 39920115). In response to plitidepsin, cells upregulate EIF2AK3 and proteins that reduce translation, but also proteins that support proteostasis via ribosome synthesis and cap-independent translation by eIF4G2 and IGF2BP2. This allows protein synthesis via m6A- mediated translation, which is insensitive to the drug. This work showed the broad-spectrum activity of plitidepsin in the replication of many viruses that rely on IRES or cap-dependent translation, and how others such as HIV-1 that can replicate via m6A alternative routes and scape the antiviral activity of this drug.

2. In Fig. 1 and Supp. Fig 1, it should be stressed that all experimental set ups performed by the authors are done at conditions in which plitidepsin cannot fully inhibit viral replication (i.e, at 10nM plaques are reduced up to 60 % and at 20nM plaques are reduced by 80%, but there is no full suppression). This could explain why clusters are not fully separated, (i.e, one infected cell replica treated with plitidepsin clusters with infected cells).

I think this is particularly relevant to understand the context in which authors are drawing conclusions, because if plitidepsin fully blocks nucleocapsid synthesis, the downstream effect on transcription would never be observed, a key aspect that needs to be clarified. Data presented herein support a model in which the main MoA of plitidepsin at fully inhibiting concentrations would be via inhibition of translation, while at sub-effective doses used in this study there is translation of N protein and a bystander antiviral effect via pro-inflammatory cytokine transcription that also operates. This is a point that should be discussed and clearly explained in the manuscript that is currently missing.

To put this in context, author could also show viral proteins differentially expressed in the proteomic datasets compared in Figure 1 and Supp. Fig 2.
Are specific viral proteins such as the nucleocapsid detected when adding plitidepsin at 10 or 20 nM in each of the variants used for infection?

Also, when authors write "Analysis of gene expression confirmed that SARS-CoV-2 infection strongly impacts Pol II transcription and immune pathways, and that Plitidepsin specifically inhibits these effects" it should be noted that the same effect will be observed for any other antiviral blocking viral replication, so the idea of "specificity" should be toned down.

3. In Figure 3, authors perform elegant ChIP-qPCR assays with N transfected cells, but their key observation should be confirmed in SARS-CoV-2 infected cells, maybe using alternative approaches.

Would this also work using an N protein of SARS-CoV or even MERS? This is something that could be studied just by comparing aa sequences of N, and would give the manuscript a better argument to present these findings as broad-spectrum effects beyond a SARS-CoV-2 infectious setting.

4. In the discussion section, the authors state that "These observations also provide a molecular explanation for the reduced transcription of early response genes such as NFκB, a key mediator of the inflammatory cascade, as well as for the decreased levels of pro-inflammatory cytokines, including IL6, IFNβ, TNFA, observed both in the nonproductive SARS-CoV-2 infections in vitro as well as in the lungs of SARSCoV-2-infected mice treated with Plitidepsin 38." However, in this study, measurements of these factors were done using proteins, not transcripts, which is consistent with plitidepsin blocking protein translation. In addition, in this paper, the same effect is observed for influenza, which does not have the same nucleocapsid as SARS-CoV-2. Effects on influenza are therefore consistent with plitidepsin blocking de novo protein translation via cap-dependent processes, rather than influencing translation via SARS-CoV-2 N protein binding to eEF1A which is lacking in this infectious model.

A key question that authors do not address is why "SARS-CoV-2 exploits multifunctional host proteins to coordinate transcriptional rewiring of the infected cells" that would lead to an immune response and inhibit viral replication. This is somehow unexpected and deserves further contextualization.

5. Biosafety permissions requested to perform this work are missing at the M&M sections.

Minor comments:

-In page 7, bottom paragraph, this sentence is repeated twice "Having observed the co-recruitment of eEF1A1 with the viral N protein at cytokine gene promoters, we next examined the molecular basis of this interaction"

-Axis in Supp Fig 4 is missing

-Reference 16 should be placed in this sentence "Recent virus-host interactome studies and drug repurposing screens have identified host translation machinery as a key vulnerability exploited by SARS-CoV-2 13,14." and not in "In particular the viral nucleocapsid N protein interferes with host translation machinery through different proteins such as the ribosomal protein L5 and the elongation factor eEF1A 15-17."

1

Reviewer #1 (Comments to the Authors (Required)):

1) The study focuses on the role of eEF1A and its potential as a host-targeted anti-viral using transcriptomics to identify how existing treatments affect viral replication, and how this ties into the broad-spectrum activity of plitidepsin. The principal advancement being how eEF1A-1 in particular interacts with SARS-CoV's N protein, and how this interaction causes transcriptomic dysregulations that lead to the hijacking of the cell's machinery, concluding that plitidepsin works by disrupting this interaction.

2) Antiviral activities of SARS-CoV 2 variants: I don't think this section is particularly relevant for the focus of the rest of the paper. I would suggest keeping the last paragraph and citing the S1 once.
-. Differential expression profiles induced by the infection of SARS-CoV2 variants:
The whole first paragraph belongs in methods.
We moved it in the Material and Methods section.
-. Volcano plots seem a bit redundant with the use of heatmaps on the same figure; they distract from the overall narrative.
We kept in account the three points raised by the referee and meet it in a new paragraph entitled; "Transcriptional Responses to SARS-CoV-2 Variants and Their Reversal by Plitidepsin". Accordingly, (i) Fig1, Supplementary FigS1 and S2 were modified; and (ii) the first paragraph of the previous result section is now in the Methods Section.
-. The viral N protein drives host gene expression: 2D seems irrelevant considering what's shown in 2C.
We apologize for the confusion. Figure 2C shows the DNA-binding motifs identified in the promoters of all upregulated genes, whereas Figure 2D shows the percentage of upregulated genes containing each of the identified motifs. We believe that both analyses provide valuable and complementary information. To improve clarity, we have revised the description in the manuscript.
The N protein is recruited at the promoter of activated genes: There's no indication of any statistical analysis in any of these graphs. I would strongly suggest including those results in the graphs, as it'll give the results more robustness.
We added the statistical analyses to the individual graphs (or provided a supplementary table listing all P values), We thank the authors for this suggestion. Since displaying all comparisons would render the figures hard to read, we only depicted main significative statistical analysis on the figures. The whole statistical analysis is associated to the paper as supplemental material.
-. Biochemical Dissection of eEF1A1-N Protein Binding: The first sentence is duplicated.
We eliminated it.
-. Again, statistical analysis would be helpful.
See above
-. Extensive methodology details should be in the methods section.
The Materials and Methods section has been revised to provide a more detailed description of the experimental procedures and to include statistical analyses and antibody references.

-. I would suggest adding a different positive control to accompany Plitidepsin, a comparison with other antivirals in this specific context would support your thesis more rigorously.
We thank the reviewer for this valuable suggestion. While comparing plitidepsin with approved antivirals could be informative in viral replication assays, the aim of this study was mainly to investigate the molecular function of the SARS-CoV-2 N protein in reprogramming host gene expression. Our experimental model relies on ectopic N protein expression rather than viral infection. Plitidepsin targets the host factor eEF1A, which interacts with the N protein, whereas nirmatrelvir/ritonavir, remdesivir, and molnupiravir act by inhibiting viral enzymes or viral RNA replication and require active viral replication to exert their effects. Therefore, these antivirals are not appropriate mechanistic controls because they act through different mechanisms than plitidepsin and, consequently, would not be expected to reverse the N protein-induced transcriptional changes observed in our model.
We have clarified this point in the revised manuscript.

3) I would suggest rigorous editing of the text; it is repetitive and lacks proper structure.
There are multiple spelling, grammar, and punctuation errors, repetitions, and large portions of the results are occupied by explaining the methodology.
We have asked a second editing.

Reviewer #2 (Comments to the Authors (Required)):

In this manuscript, Bidon and col. present novel concepts related to the role of the nucleocapsid (N) of SARS-CoV-2, which drives a host transcriptional reprograming via eEF1A that acts in this context as a nuclear factor involved in transcription of pro-inflammatory genes. While the work offers valuable novel insights identifying a conserved mechanism by which SARS-CoV-2 reprograms host gene expression across genetically diverse viral variants, it lacks a better contextualization of prior studies and the mechanism of action exerted by plitidepsin, the drug used to block eEF1A in this paper. Key points that should be reinforced include:

1). In the introduction, the authors state that "Plitidepsin inhibits the translation machinery and has shown promising activity in clear-cell ovarian cancer 26,27, and against SARS-CoV-2 28. However, the mechanisms underlying its antiviral effects remain poorly understood".
Yet there are several studies that have gain key molecular insights explaining how plitidepsin displays its antiviral effect, that even go beyond inhibiting eEF1A:
*In the seminal study by White et al. (PMID: 33495306) it was already shown that plitidepsin was inhibiting eEF1A, having a much greater impact on subgenomic viral RNA than on genomic RNA, especially when full inhibitory concentrations of the drug were used.
*Later reports efficiently showed how plitidepsin inhibit the replication cycle by inhibiting the formation of double membrane vesicles where subgenomig RNA is formed (PMID: 35231500), affecting different SARS-CoV-2 variants with equal efficacy as shown in this manuscript.
*Recently, it has been shown that plitidepsin effectively blocks cap-dependent and IRES-mediated translation via the inhibition of the elongation factor eEF1A along with the bystander phosphorylation of eF2, which is a key regulator of protein translation initiation that is also blocked by this drug (PMID: 39920115). In response to plitidepsin, cells upregulate EIF2AK3 and proteins that reduce translation, but also proteins that support proteostasis via ribosome synthesis and cap-independent translation by eIF4G2 and IGF2BP2. This allows protein synthesis via m6A- mediated translation, which is insensitive to the drug. This work showed the broad-spectrum activity of plitidepsin in the replication of many viruses that rely on IRES or cap-dependent translation, and how others such as HIV-1 that can replicate via m6A alternative routes and scape the antiviral activity of this drug.
We thank the reviewer for this comment. We agree that our wording in the Introduction may have been ambiguous. Our intention was not to suggest that the molecular mechanism of action of plitidepsin is poorly understood. Rather, we meant that the molecular mechanisms by which the SARS-CoV-2 N protein alters host cell biology, and how targeting eEF1A with plitidepsin counteracts these effects, remain incompletely understood.
The studies cited by the reviewer provide a thorough characterization of the mechanism of action of plitidepsin through its interaction with eEF1A. However, they do not specifically address the molecular mechanisms underlying N protein-induced host cell alterations, which are the focus of the present study. Our study shows that the primary changes in gene expression occur at the transcriptional level. These transcriptional alterations could subsequently lead to impaired translation through reduced protein synthesis, in addition to the direct inhibition of translation by plitidepsin.

These references have now been cited more clearly in the text.

2. In Fig. 1 and Supp. Fig 1, it should be stressed that all experimental set ups performed by the authors are done at conditions in which plitidepsin cannot fully inhibit viral replication (i.e, at 10nM plaques are reduced up to 60 % and at 20nM plaques are reduced by 80%, but there is no full suppression). This could explain why clusters are not fully separated, (i.e, one infected cell replica treated with plitidepsin clusters with infected cells).

I think this is particularly relevant to understand the context in which authors are drawing conclusions, because if plitidepsin fully blocks nucleocapsid synthesis, the downstream effect on transcription would never be observed, a key aspect that needs to be clarified. Data presented herein support a model in which the main MoA of plitidepsin at fully inhibiting concentrations would be via inhibition of translation, while at sub-effective doses used in this study there is translation of N protein and a bystander antiviral effect via pro-inflammatory cytokine transcription that also operates. This is a point that should be discussed and clearly explained in the manuscript that is currently missing.

-. To put this in context, author could also show viral proteins differentially expressed in the proteomic datasets compared in Figure 1 and Supp. Fig 2. -. Are specific viral proteins such as the nucleocapsid detected when adding plitidepsin at 10 or 20 nM in each of the variants used for infection?

As suggested by the referee, we now present a Heatmap showing the expression levels of the viral S, E, M, and N proteins in the corresponding infected Calu-3 cells further treated or not by Plitidepsin from Experiment I. This is now in Fig.1C and Text (page 5).

-. Also, when authors write "Analysis of gene expression confirmed that SARS-CoV-2 infection strongly impacts Pol II transcription and immune pathways, and that Plitidepsin specifically inhibits these effects" it should be noted that the same effect will be observed for any other antiviral blocking viral replication, so the idea of "specificity" should be toned down.

We believe this may reflect a misunderstanding related to the phrasing. The effects of antiviral compounds on immune responses can generally be attributed, at least in part, to a reduction in overall viral load, which subsequently limits the inflammatory response associated with viral infection. In our case, however, Plitidepsin treatment significantly reverses the effects of N protein transfection on the expression of Pol II-transcribed genes, including several genes associated with immune pathways. To our knowledge, such an effect of an antiviral compound on N protein-induced transcriptional changes has not previously been described.

We modify the text accordingly (page 5).

3. In Figure 3, authors perform elegant ChIP-qPCR assays with N transfected cells, but their key observation should be confirmed in SARS-CoV-2 infected cells, maybe using alternative approaches.

We also considered the experiment suggested by the reviewer, however, it is technically and logistically very challenging. SARS-CoV-2 infection experiments must be performed in a BSL-3 facility. Preparing chromatin for ChIP-qPCR would therefore require all the chromatin preparation steps (including chromatin preparation itself, sonication, immunoprecipitation and qPCR) to be carried out inside the BSL-3 laboratory, which is not equipped for these

procedures. In addition, infected material cannot be removed from the BSL-3 laboratory without complete pathogen inactivation, a process that would disrupt chromatin integrity and transcriptional complexes, making ChIP analysis impossible. We therefore performed ChIP-qPCR using chromatin from N protein-transfected cells, which enabled us to obtain the results presented in the manuscript.

-. Would this also work using an N protein of SARS-CoV or even MERS? This is something that could be studied just by comparing aa sequences of N, and would give the manuscript a better argument to present these findings as broad-spectrum effects beyond a SARS-CoV-2 infectious setting.

In response, we compared the amino acid sequences of the nucleocapsid (N) proteins from SARS-CoV, MERS-CoV, and SARS-CoV-2 and found that the eEF1A1 interacting region is highly conserved among these coronaviruses (Supplementary Figure S5G). We have included these data and expanded the Discussion to emphasize that the conserved N–eEF1A1 interaction may represent a broad-spectrum antiviral target for pathogenic coronaviruses beyond SARS-CoV-2. (see at the end of the Discussion).

4. In the discussion section, the authors state that "These observations also provide a molecular explanation for the reduced transcription of early response genes such as NFκB, a key mediator of the inflammatory cascade, as well as for the decreased levels of pro-inflammatory cytokines, including IL6, IFNβ, TNFA, observed both in the nonproductive SARS-CoV-2 infections in vitro as well as in the lungs of SARSCoV-2-infected mice treated with Plitidepsin 38." However, in this study, measurements of these factors were done using proteins, not transcripts, which is consistent with plitidepsin blocking protein translation.

We also cannot exclude a role of N in modulating translation. Indeed, when recombinant N-WT protein was added to an in vitro translation assay containing rabbit reticulocyte lysate, endogenous translation factors, capped luciferase RNA, and the other components required for translation, N-WT promoted luciferase translation (Annexe Figure A). When N-WT protein was maintained at a fixed concentration, Plitidepsin produced a dose-dependent inhibition of this N-WT-associated translational activity.

However, the molecular mechanism underlying this effect remains difficult to dissect in this experimental system. In particular, we cannot determine whether the inhibitory effect of Plitidepsin results from disruption of the N/eEF1A1 interaction during translation, impaired recruitment of the N/eEF1A1 complex to the translation machinery, direct inhibition or functional inactivation of eEF1A1 itself, or a combination of these effects. Thus, while these results support an effect of Plitidepsin on N-associated translational activity, they do not allow us to assign the inhibitory effect to a specific molecular mechanism.

[Figure removed by editorial staff per authors' request].

-. In addition, in this paper, the same effect is observed for influenza, which does not have the same nucleocapsid as SARS-CoV-2. Effects on influenza are therefore consistent with plitidepsin blocking de novo protein translation via cap-dependent processes, rather than influencing translation via SARS-CoV-2 N protein binding to eEF1A which is lacking in this infectious model.

While the effect of Plitidepsin on H1N1 induced immune genes seems much weaker than for COVID, there is certainly still a translational effect of Plitidepsin over the expression of cytokine. Structural studies have shown that plitidepsin binds to the C-terminal domain (CTD) of eEF1A1. In the present paper, we demonstrated that plitidepsin disrupts the interaction between the eEF1A1 CTD and the SARS-CoV-2 N protein.

However, in our in vitro translation assays, it was difficult to distinguish whether plitidepsin was directly impairing the intrinsic translation function of eEF1A1 (thereby affecting host protein synthesis) or specifically preventing the stimulatory effect of the N protein on translation. However, an additional role of Plididepsin in disrupting eEF1A1 when involved in translation cannot be excluded.

We did not find any similarities between NCAP-SARS2 & NCAP-H1N1.

-. A key question that authors do not address is why "SARS-CoV-2 exploits multifunctional host proteins to coordinate transcriptional rewiring of the infected cells" that would lead to an immune response and inhibit viral replication. This is somehow unexpected and deserves further contextualization.

We thank the reviewer for this important comment. Although eEF1A1 is best known for its canonical role in translation, it is a multifunctional protein with additional functions in the regulation of transcription, cytoskeletal organization, and other cellular processes. Our study focuses on this non-canonical role (at the transcriptional level) of eEF1A1. We first show that eEF1A1 is involved in the formation of a transcription activation process, and show (using ChiP) that interaction of the SARS-CoV-2 N protein with eEF1A1 contributes to transcriptional reprogramming of the host cel. Such transcriptional changes reflect the consequences of perturbing the regulatory functions of eEF1A1 in gene expression, rather than a direct effect on the translational machinery.

We have revised the manuscript to better emphasize this distinction by writing "However, these studies have primarily linked eEF1A to viral translation (Supplemental Figure 6) or RNA replication. Our results extend this paradigm by demonstrating that the SARS-CoV-2 nucleocapsid protein recruits eEF1A1 to cytokine gene promoters together with transcription factors and Pol II, thereby revealing an unexpected role for eEF1A1 in transcriptional regulation."

5. Biosafety permissions requested to perform this work are missing at the M&M sections.
All SARS-CoV-2 experiments were performed in the certified biosafety laboratory of the College of Medicine of the NTU by trained personnel authorized to handle SARS-CoV-2 strains. These investigators are co-authors of the manuscript.
The Materials and Methods section has been revised to clarify the biosafety conditions under which these experiments were conducted.
-. Minor comments:
In page 7, bottom paragraph, this sentence is repeated twice "Having observed the co-recruitment of eEF1A1 with the viral N protein at cytokine gene promoters, we next examined the molecular basis of this interaction"
-Axis in Supp Fig 4 is missing
The manuscript have been modified accordingly.
-**Reference 16** should be placed in this sentence "Recent virus-host interactome studies and drug repurposing screens have identified host translation machinery as a key vulnerability exploited by SARS-CoV-2 13,14." and not in "In particular the viral nucleocapsid N protein interferes with host translation machinery through different proteins such as the ribosomal L5 and the elongation factor eEF1A1 15-17.
References were modified.

September 14, 2026

RE: Life Science Alliance Manuscript #LSA-2026-03751-TR

Jean-Marc Egly
IGBMC
France

Dear Dr. Egly,

Thank you for submitting your revised manuscript entitled "Hijacking Host Transcription by targeting eEF1A1: Novel Function of SARS-CoV-2 Nucleocapsid Protein." As you will see, both reviewers are satisfied overall. We direct your attention to the minor issues noted by Reviewer 2. We would be happy to publish your paper in Life Science Alliance pending final revisions necessary to meet our formatting guidelines.

MANUSCRIPT ORGANIZATION AND FORMATTING:

To avoid unnecessary delays in the acceptance and publication of your paper, please read the following information carefully. Full guidelines are available on our Instructions for Authors page, https://www.life-science-alliance.org/authors

Submission of a paper that does not conform to Life Science Alliance guidelines will delay the acceptance of your manuscript.

-We suggesting changing the manuscript title to "SARS-CoV-2 Nucleocapsid Protein Hijacks Host Transcription by targeting eEF1A1"
-Please upload your Tables in editable .doc or Excel format; They should be labeled as Table 1 and, for supplementary tables, as Table S1, S2, etc., not as Extended data.
-Please add the ORCID ID for the corresponding author--you should have received instructions on how to do so.
-Please add Keywords for your manuscript in our system.
-Please add the X and Bluesky handles of your host institute/organization, as well as your own, and/or one of the authors, in our system.
-Please be sure that the authorship listing and order are correct and match between the system and manuscript file.
-Please be sure that all authors are mentioned in the authors' Contribution section in the manuscript file.
-Please rename "Disclosure and competing interest's statement" to "Conflict of Interest".
-Please be sure to add call-outs for your supplementary tables (for instance in the methods section) and also to add their legends below the supplementary figure legends in the manuscript text.
-Please add callouts for Figures 2D and S3A-C to your main manuscript text.
-Please provide full details for the cytokine quantification protocol in the methods section. Please also indicate the working concentration of antibodies used.
-Please indicate replicates, variance measures (i.e. standard deviation), as well as statistical tests in the figure legends for all figure panels depicting summary data (e.g. Figures 3 and 4, Supplementary Figure 1, etc.).

We encourage our authors to provide original source data, particularly uncropped/-processed electrophoretic blots and spreadsheets for the main figures of the manuscript. If you would like to add source data, we would welcome one PDF/Excel-file per figure for this information. These files will be linked as supplementary "Source Data" files.

It is Life Science Alliance policy that if requested, original data images must be made available to the editors. Failure to provide original images upon request will result in unavoidable delays in publication. Please ensure that you have access to all original data images prior to final submission.

If you are planning a press release on your work, please inform us immediately to allow informing our production team and scheduling a release date.

We welcome submissions of potential cover images for the issue of LSA in which your work would appear. If you have high quality images associated with this work, please feel free to email these, with a caption, to the journal office.

LSA encourages authors to provide a 30-60 second video where the study is briefly explained. These videos will be appear embedded with the manuscript online at Life Science Alliance, and on social media to promote the published paper and authors (for examples, see https://docs.google.com/document/d/1-UWCfbE4pGcDdcgzcmiuJl2XMBJnxKYeqRvLLrLSo8s/edit?usp=sharing). Corresponding or first-authors are welcome to submit the video. Please submit only one video per manuscript.

The video can be emailed to contact@life-science-alliance.org

FINAL FILES:

To upload the final version of your manuscript, please log in to your account: https://lsa.msubmit.net/cgi-bin/main.plex
You will be guided to complete the submission of your revised manuscript and to fill in all necessary information. Please get in touch in case you do not know or remember your login name.

The following items are required for acceptance.

-- An editable version of the final text (.DOC or .DOCX) is needed for copyediting (no PDFs).

-- High-resolution figure, supplementary figure and video files uploaded as individual files: See our detailed guidelines for preparing your production-ready images, https://www.life-science-alliance.org/authors

-- Summary blurb (enter in submission system): A short text summarizing in a single sentence the study (max. 200 characters including spaces). This text is used in conjunction with the titles of papers, hence should be informative and complementary to the title. It should describe the context and significance of the findings for a general readership; it should be written in the present tense and refer to the work in the third person. Author names should not be mentioned.

The license to publish form must be signed before your manuscript can be sent to production. A link to the license to publish form will be available to the corresponding author only. Please take a moment to check your funder requirements.

Reviews, decision letters, and point-by-point responses associated with peer-review at Life Science Alliance will be published, alongside the manuscript. If you do want to opt out of having the reviewer reports and your point-by-point responses displayed, please let us know immediately.

Thank you for your attention to these final processing requirements. Please revise and format the manuscript and upload materials as soon as you are able.

Thank you for this interesting contribution to the literature. We look forward to publishing your paper in Life Science Alliance.

Sincerely,

Tim Fessenden
Scientific Editor
Life Science Alliance

Reviewer #1 (Comments to the Authors (Required)):

1. The study focuses on the role of eEF1A and its potential as a host-targeted anti-viral using transcriptomics to identify how existing treatments affect viral replication, and how this ties
into the broad-spectrum activity of plitidepsin. The principal advancement being how eEF1A-1 in particular interacts with SARS-CoV's N protein, and how this interaction causes transcriptomic dysregulations that lead to the hijacking of the cell's machinery, concluding that plitidepsin works by disrupting this interaction.
2. I consider the responses sufficiently resolved.
3. I consider the responses sufficiently resolved.

Reviewer #2 (Comments to the Authors (Required)):

The authors have addressed all my comments, and the manuscript can be now accepted.
There are however some typos that I found while reviewing the new version of the manuscript that should be changed before publishing the paper:

-Figure 1A. Panel says Plitidepsin 10 uM and 20 uM instead of nM in both cases. Please correct.
-Figure 1B. Panel says Plitidepsin 10 uM instead of nM. Please correct.
-Legend of figure 1: White and black boxes indicate Inf and Inf+Pliti, when it should read control and Pliti, as Mock cells are equally labeled but not infected.
-Supplemental Figure 2 B. Panel says Plitidepsin 10 uM and 20 uM instead of nM in both cases. Please correct.

-Figure 2 D is not called in the main text.
-Supplemental Figure 3A and B do not have an X-axis in the figure, which I guess should be "gene count". Please add that label.
-Legend of supplemental figure 3: White and black boxes indicate Inf and Inf+Pliti, when it should read control and Pliti, as Mock cells they are equally labeled but not infected.
-Supplemental Figure 4A does not have an X-axis in the figure, which I guess should be "gene count". Please add that label.
-Supplemental Figure 5G does not have an explanation or call in the main result text, only in the discussion.

September 24, 2026

RE: Life Science Alliance Manuscript #LSA-2026-03751-TRR

Prof. Jean-Marc Egly
Institut de génétique et de biologie moléculaire et cellulaire
France

Dear Dr. Egly,

Thank you for submitting your Research Article entitled "SARS-CoV-2 Nucleocapsid protein hijack host transcription by targeting eEF1A1.". It is a pleasure to let you know that your manuscript is now accepted for publication in Life Science Alliance. Congratulations on this interesting work!

The final published version of your manuscript will be deposited by us to PubMed Central upon publication.

Your manuscript will now progress through copyediting and proofing. During the proofing stage, kindly correct the title ("hijacks host transcription") and ensure that working concentrations of antibodies are indicated in the methods section.

Your article will publish open access upon publication under a CC-BY license.

Reviews, decision letters, and point-by-point responses associated with peer-review at Life Science Alliance will be published, alongside the manuscript. If you do want to opt out of having the reviewer reports and your point-by-point responses displayed, please let us know immediately.

IMPORTANT: If you will be unreachable at any time, please provide us with the email address of an alternate author. Failure to respond to routine queries may lead to unavoidable delays in publication.

Scheduling details will be available from our production department. You will receive proofs shortly before the publication date. Only essential corrections can be made at the proof stage so if there are any minor final changes you wish to make to the manuscript, please let the journal office know now.

DISTRIBUTION OF MATERIALS:
Authors are required to distribute freely any materials used in experiments published in Life Science Alliance. Authors are encouraged to deposit materials used in their studies to the appropriate repositories for distribution to researchers.

You can contact the journal office with any questions, contact@life-science-alliance.org

Again, congratulations on a very nice paper. I hope you found the review process to be constructive and are pleased with how the manuscript was handled editorially. We look forward to future exciting submissions from your lab.

Sincerely,

Tim Fessenden
Scientific Editor
Life Science Alliance
