# Supplementary material for: SARS-CoV-2 nucleocapsid protein hijacks host transcription by targeting eEF1A1
Source: Life Sci Alliance. 2026 Oct 14;9(12):e202603751. doi: 10.26508/lsa.202603751 (PMC13669674; doi:10.26508/lsa.202603751)
Supplement: Supplementary file 2 [file LSA-2026-03751_TableS1.docx]

| Plasmid | Vector | Vector restriciton | Insert restriction | Primer sequences |
| --- | --- | --- | --- | --- |
| GFP-N-FL | pEGFP-C1 | BglII, SalI | BamHI, SalI | 5’-TAAGGATCCATGTCTGATAATGGACCCCAAAATC  5’-TAAGTCGACTTAGGCCTGAGTTGAGTCAGCAC |
| GFP-N1-174 | pEGFP-C1 | BglII, SalI | BamHI, SalI | 5’-TAAGGATCCATGTCTGATAATGGACCCCAAAATC  5’-TAAGTCGACTTCTGCGTAGAAGCCTTTTGGC |
| GFP-N1-254 | pEGFP-C1 | BglII, SalI | BamHI, SalI | 5’-TAAGGATCCATGTCTGATAATGGACCCCAAAATC  5’-TAAGTCGACAGCCTCAGCAGCAGATTTCTTAGTG |
| GFP-N1-364 | pEGFP-C1 | BglII, SalI | BamHI, SalI | 5’-TAAGGATCCATGTCTGATAATGGACCCCAAAATC  5’-TAAGTCGACTGGGAATGTTTTGTATGCGTC |
| GFP-N174-254 | pEGFP-C1 | BglII, SalI | BamHI, SalI | 5’-TAAGGATCCGGGAGCAGAGGCGGC  5’-TAAGTCGACAGCCTCAGCAGCAGATTTCTTAGTG |
| GFP-N255-419 | pEGFP-C1 | BglII, SalI | BamHI, SalI | 5’-TAAGGATCCTCTAAGAAGCCTCGGCAAAAAC  5’-TAAGTCGACTTAGGCCTGAGTTGAGTCAGCAC |
| GFP-NΔ355-363 | pEGFP-C1 | BglII, SalI | BamHI, SalI | 5’-TAAGGATCCATGTCTGATAATGGACCCCAAAATC  5’-GCTCTGTTGGTGGATTCAGCAAAATGACTTGATCTTTG  5’-CATTTTGCTGAATCCACCAACAGAGCCTAAAAAGG  5’-TAAGTCGACTTAGGCCTGAGTTGAGTCAGCAC |
| Flag-N-FL | pCMV-Tag2B | BamHI, XhoI | BamHI, SalI | 5’-TAAGGATCCATGTCTGATAATGGACCCCAAAATC  5’-TAAGTCGACTTAGGCCTGAGTTGAGTCAGCAC |
| Flag-NΔ355-363 | pCMV-Tag2B | BamHI, XhoI | BamHI, SalI | 5’-TAAGGATCCATGTCTGATAATGGACCCCAAAATC  5’-GCTCTGTTGGTGGATTCAGCAAAATGACTTGATCTTTG  5’-CATTTTGCTGAATCCACCAACAGAGCCTAAAAAGG  5’-TAAGTCGACTTAGGCCTGAGTTGAGTCAGCAC |
| GFP-eEF1A1 | pEGFP-C1 | BglII, SalI | BamHI, XhoI | 5’-TAAGGATCCATGGGAAAGGAAAAGACTCATATC  5’-ACTCGAGTCATTTAGCCTTCTGAGCTTTC |
| GFP-  eEF1A11-240 | pEGFP-C1 | BglII, SalI | BamHI, XhoI | 5’-TAAGGATCCATGGGAAAGGAAAAGACTCATATC  5’-ACTCGAGACGAGTTGGTGGTAGGATG |
| GFP-  eEF1A11-335 | pEGFP-C1 | BglII, SalI | BamHI, XhoI | 5’-TAAGGATCCATGGGAAAGGAAAAGACTCATATC  5’-ACTCGAGCATTGGTGGGTCATTTTTG |
| GFP-  eEF1A11-387 | pEGFP-C1 | BglII, SalI | BamHI, XhoI | 5’-TAAGGATCCATGGGAAAGGAAAAGACTCATATC  5’-ACTCGAGCAGCTTTTTACCAGAACGGC |
| GFP-  eEF1A11-437 | pEGFP-C1 | BglII, SalI | BamHI, XhoI | 5’-TAAGGATCCATGGGAAAGGAAAAGACTCATATC  5’-ACTCGAGGACACCCACCGCAACT |
| GFP-  eEF1A1241-355 | pEGFP-C1 | BglII, SalI | BamHI, XhoI | 5’-TAAGGATCCCCAACTGACAAGCCCTTG  5’-ACTCGAGCATTGGTGGGTCATTTTTG |
| GFP-  eEF1A1356-462 | pEGFP-C1 | BglII, SalI | BamHI, XhoI | 5’-TAAGGATCCGAAGCAGCTGGCTTCAC  5’-ACTCGAGTCATTTAGCCTTCTGAGCTTTC |
| Flag-eEF1A1 | pCMVTag2B | BamHI, XhoI | BamHI, XhoI | 5’-TAAGGATCCATGGGAAAGGAAAAGACTCATATC  5’-ACTCGAGTCATTTAGCCTTCTGAGCTTTC |
| Flag-eEF1A2 | pCMVTag2B | BamHI, XhoI | BglII, XhoI | 5’-AAAGATCTATGGGCAAGGAGAAGACC  5’-ACTCGAGTCACTTGCCCGCCTT |
