# Supplementary material for: SARS-CoV-2 nucleocapsid protein hijacks host transcription by targeting eEF1A1
Source: Life Sci Alliance. 2026 Oct 14;9(12):e202603751. doi: 10.26508/lsa.202603751 (PMC13669674; doi:10.26508/lsa.202603751)
Supplement: Supplementary file 3 [file LSA-2026-03751_TableS2.docx]

| RNA expression | Primer sequences |
| --- | --- |
| IFNb | 5’- GCACAACAGGTAGTAGGCGA  5’- AGAAGCACAACAGGAGAGCA |
| IL6 | 5’- ACTCACCTCTTCAGAACGAATTG  5’- CCATCTTTGGAAGGTTCAGGTTG |
| OASL | 5’- CCATTGTGCCTGCCTACAGAG  5’- CTTCAGCTTAGTTGGCCGATG |
| FRG | 5’- AATTGGGCTCCAGGTCTCTG  5’- CACCGGCTCCAATTTCTTGC |
| IFNd | 5’- CTTCCAAGCCCACCCCAACT  5’- GGCCTCCAGGACCTTCAGC |
| TGFb | 5’- ACTATTGCTTCAGCTCCACG  5’- TTATGCTGGTTGTACAGGGC |
| ChIP | Primer sequences |
| IFNb-prox | 5’- GGCCTATTTATATGAGATGGTCCT  5’- TGACATAGGAAAACTGAAAGGGAGA |
| IFNb-distal | 5’- AGTCCCTGCACCAGTTCCTT  5’- GACAAATTGGCTCCTATCTCTGAAA |
| IL6-prox | 5’- TCGTGCATGACTTCAGCTTTAC  5’- GGCTAGCGCTAAGAAGCAGAA |
| IL6-distal | 5’- TGGTTCAGGAGTGTCTACGTT  5’- CATAACATTTCAGGACCCGCC |
| OASL-prox | 5’- GGCTGGCCGTCTAGTCAATC  5’- CAAGTTGCCTCCTTTGGGGT |
| OASL-distal | 5’- AGAGTGGGTTTAACAGGTATTATGA  5’- GCACTGGACTTTTGCAGTCTC |
| FRG-prox | 5’- AGAGTGGGTTTAACAGGTATTATGA  5’- GCACTGGACTTTTGCAGTCTC |
